# Long‐term outcomes following alternative second‐line oral glucose‐lowering treatments: Results from the real‐world progression in type 2 diabetes mellitus United Kingdom (RAPIDS‐UK) model

**DOI:** 10.1111/dom.16447

**Published:** 2025-05-21

**Authors:** Orlagh U. Carroll, Patrick Bidulka, Anirban Basu, Amanda I. Adler, Stephen O'Neill, Andrew H. Briggs, David G. Lugo‐Palacios, Kamlesh Khunti, Richard Grieve

**Affiliations:** ^1^ Department of Health Services Research and Policy London School of Hygiene & Tropical Medicine London UK; ^2^ Department of Non‐Communicable Disease Epidemiology London School of Hygiene & Tropical Medicine London UK; ^3^ The Comparative Health Outcomes, Policy & Economics (CHOICE) Institute University of Washington School of Pharmacy Seattle Washington USA; ^4^ Diabetes Trials Unit, The Oxford Centre for Diabetes, Endocrinology and Metabolism University of Oxford, OCDEM Building Churchill Hospital Headington UK; ^5^ Diabetes Research Centre University of Leicester Leicester UK

**Keywords:** diabetes complications, effectiveness, health economics, real‐world evidence, SGLT2 inhibitor, type 2 diabetes

## Abstract

**Aims:**

To compare long‐term complications for people with type 2 diabetes mellitus (T2DM) following second‐line treatment in routine practice with sulphonylureas (SU), dipeptidyl peptidase‐4 inhibitors (DPP4i), or sodium‐glucose co‐transporter‐2 inhibitors (SGLT2i) added to metformin.

**Materials and Methods:**

We used the RAPIDS microsimulation model to predict diabetes complications over 5 years after second‐line treatment initiation. We combined information on ‘real‐world’ treatment duration in England from the Clinical Practice Research Datalink with evidence on treatment effectiveness from Randomised Controlled Trials (RCTs). We estimated between‐treatment differences in the probabilities of end‐stage kidney disease (ESKD), heart failure hospitalisation (HF), diabetic eye disease, myocardial infarction (MI), and lower‐extremity amputation (LEA).

**Results:**

The predicted probabilities of complications within 5 years were lower following second‐line treatment with SGLT2i compared to SU and DPP4i. The mean (95% CI) difference (reduction) in the predicted probability of ESKD following SGLT2i versus SU was −0.81% (−0.89, −0.73), and for SGLT2i versus DPP4i the corresponding difference was −0.87% (−0.95, −0.79). The reduction in the probability of HF following SGLT2i versus SU was −0.90% (−1.01, −0.80), and for SGLT2i versus DPP4i it was −0.95% (−1.06, −0.84). The corresponding differences in the probabilities of diabetic eye disease following SGLT2i versus SU were −1.41% (−1.57, −1.26), and for SGLT2i versus DPP4i was −0.44% (−0.59, −0.29). The predicted probabilities of LEA were similar across treatments. Pre‐existing CVD did not modify the predicted probabilities of complications.

**Conclusions:**

For a general T2DM population, second‐line treatment with SGLT2i rather than SU or DPP4i can reduce the probability of complications within 5 years.

## 1 INTRODUCTION

For people with type 2 diabetes mellitus (T2DM), multifactorial interventions improving glycated haemoglobin A1c (HbA1c), blood pressure, and lipids can reduce the risk of long‐term complications.^1^, ^2^ If glycaemic control is inadequate after metformin monotherapy, second‐line oral glucose‐lowering treatments are recommended.^3^, ^4^ For those with pre‐existing cardiovascular disease (CVD), or at high risk of CVD, heart failure, or kidney disease, SGLT2i are recommended,^3^, ^4^ but for most people with T2DM, an international consensus statement^4^ and NICE guidelines^3^ leave the choice of second‐line treatment to clinicians and patients. Globally, the most prevalent second‐line treatments are: dipeptidyl peptidase‐4 inhibitors (DPP4i) (48.3%), sulphonylureas (SU) (40.9%), and sodium‐glucose co‐transporter‐2 inhibitors (SGLT2i) (8.3%), added to metformin.^5^

Randomised controlled trials (RCTs) have established that second‐line treatment with SGLT2i is generally safe and efficacious for people with T2DM,^6^, ^7^, ^8^, ^9^ but using these findings is challenging because trial participants may differ to those who present for treatment in routine practice. Also, published RCTs and meta‐analyses do not provide a full assessment of the impact that alternative second‐line treatments have on all long‐term complications including diabetic eye disease (retinopathy and/or blindness) and lower extremity amputation (LEA).^10^, ^11^, ^12^

Microsimulation models can address this gap in evidence by predicting the long‐term impact of alternative treatments on a full range of diabetes complications according to individuals' characteristics, treatment patterns, and subsequent events. Microsimulations have not previously been used to assess the comparative effectiveness of second‐line treatment with SGLT2i for people with T2DM.^13^, ^14^, ^15^, ^16^ The Real‐World Progression in Diabetes (RAPIDS) model is a microsimulation originally developed with US Veterans Affairs data, and recently calibrated on Medicare and Medicaid claims data.^17^ The advantage of this model is that it recognises that glucose‐lowering treatments for people with T2DM change over time,^14^ and that treatments at different places in the treatment pathway (second‐line, third‐line, etc.) may have a direct effect on complication risks (e.g. diabetic eye disease), or an indirect effect via intermediate risk factors (e.g. HbA1c). The RAPIDS model also recognises that there might be differences between RCTs and routine practice in the baseline characteristics of people presenting for treatment.

The aim of this paper is to predict the probabilities of long‐term complications for people with T2DM in England following alternative second‐line glucose‐lowering treatment in routine practice.

## 2 METHODS

### 2.1 Study overview

**FIGURE 1:**
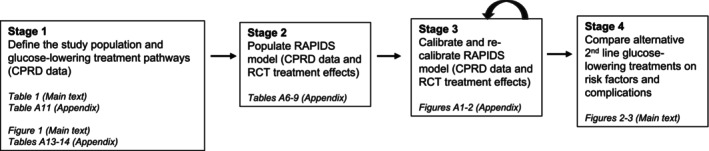
Study schematic.

We used the RAPIDS model to predict the impact of alternative second‐line glucose‐lowering treatments on risk factors and outcomes. Figure 1 provides a study schematic. We used information from the PERMIT study to define the study population and treatment pathway (*stage one*) (Table 1, Appendix section 1, and Table A1). We then populated the RAPIDS model with this information from the PERMIT study on the population and treatment pathways^18^, ^19^ and from published meta‐analyses and RCTs^20^, ^21^, ^22^, ^23^, ^24^ on the treatment effects of the alternative treatments on risk factors and complications (*stage two*). As there may be cultural, behavioural, or clinical differences between the eligible US populations for which the RAPIDS model was developed, and the UK population of interest (Table A2),^25^ we assessed the accuracy of the model's predictions versus the risk factors and complications recorded on the Clinical Practice Research Datalink (CPRD) for a cohort of people with T2DM who had second‐line treatment as part of routine practice in England. We recalibrated the model; that is, where necessary, we adjusted the RAPIDS model to accurately predict risk factors and complications in the CPRD data (*stage three*).^14^

**TABLE 1:** Baseline characteristics of the England primary‐secondary care linked study population.

Characteristics	Total
	*N* = 62 640
Age (years), median (IQR)	60.0 (52.0–69.0)
Female, count (%)	24 703 (39.4)
Ethnicity, count (%)	
White	45 396 (72.5)
Black	3125 (5.0)
Hispanic	0 (0.0)
Other (South Asian, Mixed, Other, missing)	14 119 (22.5)
Index of multiple deprivation (IMD), count (%)	
1 (least deprived)	8866 (14.2)
2	11 098 (17.7)
3	11 330 (18.1)
4	13 729 (21.9)
5 (most deprived)	14 502 (23.2)
Missing	3115 (5.0)
Years since diagnosis with T2DM, median (IQR)	4.9 (2.5–5.0)
BMI (kg/m^2^), median (IQR)	31.3 (28.0–36.0)
Systolic blood pressure (mm Hg), median (IQR)	132 (124–140)
Diastolic blood pressure (mm Hg), median (IQR)	79.0 (73.0–82.0)
HDL (mmol/L), median (IQR)	42.5 (38.7–50.3)
LDL (mmol/L), median (IQR)	81.2 (61.9–104.4)
Total cholesterol (mmol/L), median (IQR)	158.5 (139.2–185.6)
Triglycerides, non‐fasting (mmol/L), median (IQR)	159.4 (115.1–221.4)
eGFR (mL/min/1.73 m^2^), median (IQR)	92 (80–102)
HbA1c (%), median (IQR)	7.9 (7.3–8.8)
HbA1c (mmol/mol) median (IQR)	63 (56–73)
History of any CVD^a^, count (%)	8642 (13.8)
History of angina, count (%)	2053 (3.3)
History of myocardial infarction, count (%)	3820 (6.1)
History of stroke, count (%)	2621 (4.2)
History of heart failure	2910 (4.7)
History of hypoglycaemia, count (%)	491 (0.8)

^a^ CVD is a composite of angina, myocardial infarction, stroke, and heart failure.

Finally, we used the recalibrated model (RAPIDS‐UK) to compare the predicted risk factors and complications across the alternative second‐line treatments over a maximum follow‐up of 7.6 years (*stage four*). We chose this duration of follow‐up because the numbers with available data declined over time (Table A3), and after this timepoint the model provided less accurate predictions for some risk factors.

### 2.2 Stage 1: Defining the population and treatment pathways

#### 2.2.1 Population

We defined the population of interest from the PERMIT study which used primary care data from the publicly‐funded healthcare system in England, obtained from the CPRD (about 20% of the UK population),^26^, ^27^ linked to secondary care data (Hospital Episode Statistics (HES)),^28^ and death data (Office of National Statistics).^29^ The PERMIT study defined an eligible cohort of people with T2DM aged 18 years and over, who had a first‐ever prescription of SU, DPP4i, or SGLT2i, added to metformin monotherapy (see Appendix Stage 1 and Table A1). We extracted information from the PERMIT study to define baseline characteristics for people with T2DM presenting for second‐line treatment.^18^, ^19^

#### 2.2.2 Treatment pathways

The RAPIDS model also required information on individual patients' treatment pathways (second‐line, and subsequent treatments), risk factors, and complications. We extracted information from PERMIT on the duration each patient was prescribed second‐line treatment (metformin‐SU, metformin‐DPP4i, or metformin‐SGLT2i). We recognised that people could ‘add’, ‘switch’ or ‘remove’ drugs, including insulin, across the follow‐up period. We collated data on which class of subsequent glucose‐lowering therapies each patient was prescribed and how long for. The drug classes included thiazolidinediones (TZD), insulin, glucagon‐like peptide‐1 receptor‐agonists (GLP1‐RA), glinides, and acarbose. We extracted information on prescriptions for the main concomitant treatments (statins, and blood pressure‐lowering drugs).

### 2.3 Stage 2: Populating and implementing the RAPIDS model

#### 2.3.1 Overview

We populated the RAPIDS model with the above PERMIT data on individuals' baseline characteristics and treatment patterns over time. We used the RAPIDS model risk equations that summarised treatment‐specific relationships between patient characteristics, risk factors, and complications to predict diabetes‐related risk factors and complications over time. The risk factors were: HbA1c; body mass index (BMI); serum HDL, LDL and total cholesterol; serum triglycerides; systolic blood pressure (SBP); diastolic blood pressure (DBP) and estimated glomerular filtration rate (eGFR).^14^ The complications were: hypoglycaemia, myocardial infarction (MI), unstable angina, stroke, heart failure hospitalisation (HF), LEA, ESKD, diabetic eye disease (either diabetic retinopathy and/or blindness), and all‐cause death (Table A4). We also defined a subgroup of interest according to pre‐existing CVD status (Table A5).

#### 2.3.2 Treatment effects

The RAPIDS model incorporated effects of each antidiabetic treatment on each risk factor and diabetes complication (see Tables A6–A9). We considered the treatment classes present within the CPRD cohort at any time during the follow‐up period. The treatment classes prescribed after second‐line treatment were: (1) no therapy; (2) monotherapies including: metformin, SU, DPP4i, SGLT2i, GLP1‐RA, TZD, and insulin; (3) dual therapies that were different from the patient's second‐line treatment including: metformin‐SU, metformin‐DPP4i, metformin‐SGLT2i, metformin‐GLP1‐RA, metformin‐TZD, metformin‐insulin, SU‐TZD, SU‐insulin, and TZD‐insulin; (4) triple therapies and any other monotherapies or combinations of glucose‐lowering treatments (2‐, 4‐, 5‐, and 6‐drug combination therapies) which were all grouped into a single category.

We used the estimated effects of each drug class compared to no treatment on risk factors and diabetes complications from published meta‐analyses and RCTs^20^, ^21^, ^22^, ^23^, ^24^ as reported in the recently published RAPIDS (2.0) model^17^ (see also Appendix Stage 2 and Tables A6–A9).

#### 2.3.3 Implementation

We defined ‘baseline’ as just prior to initiating second‐line treatment. Each patient's subsequent pathway over the 7.6 years follow‐up period within the model was split into 90‐day ‘quarters’. We used the RAPIDS model's risk equations to predict each person's risk factors and complications during each quarter. For acute complications (hypoglycaemia, MI, unstable angina, stroke), a patient could experience more than one such event, and the probability of that event reflected whether it was a first or subsequent event.

We defined the glucose‐, cholesterol‐ and blood pressure‐lowering therapies received within each quarter from the prescription information within the CPRD data. We predicted risk factors and complications from second‐line treatment initiation until the observed data were censored due to no further CPRD data, all‐cause death, or the end of the prediction period (maximum of 7.6 years, 31 December 2020). We took average predictions across all individuals for all endpoints at each time point, along with 95% normal‐based confidence intervals (CI) of the averaged predicted values across the sample.

### 2.4 Stage 3: Calibrating and recalibrating the model

We calibrated the RAPIDS model predictions to the CPRD data (see Appendix Stage 3, Table A10, Figures A1 and A2). For each risk factor and complication, we compared the predicted values in each quarter to the corresponding ‘observed’ values from an independent hold‐out sample of the CPRD cohort that were not used in the model development (Table A10). We defined ‘agreement’ as when the observed values were within the 95% predicted intervals. For most risk factors and all complications, the observed values fell within the 95% CI of the predictions from the RAPIDS model across the full follow‐up period. For BMI, total cholesterol, eGFR and DBP (after 7.6 years) the observed values were outside the predicted 95% CI (Figure A1). We therefore undertook re‐calibration, in that we adjusted the risk equations for these outcomes. Following this recalibration, the observed values for total cholesterol were within the prediction intervals, and for DBP and BMI the remaining discrepancies between the predicted and observed values were small and so no further recalibration was undertaken (Figure A2).

### 2.5 Stage 4: Comparison of alternative second‐line treatment on risk factors and complications (see also appendices, stage 4)

For each person, we used the recalibrated model (RAPIDS‐UK) to predict three sets of model outcomes (risk factors and complications), one following each second‐line treatment (SGLT2i, DPP4i, or SU). These ‘counterfactual outcomes’ refer to each individual's predicted (hypothetical) outcomes after initiation of each second‐line treatment, recognising that an individual's actual outcome can only be observed after one of the second‐line treatments. We predicted counterfactual outcomes by defining three identical copies of each individual's observed baseline characteristics, duration of second‐line treatment received, and subsequent treatments. We assumed the individual's duration of second‐line treatment was the same in predicting each of the three counterfactual outcomes. In generating each of the three counterfactual outcomes, we also assumed that each person followed the same treatment pathway as in their observed data. These assumptions were supported by the PERMIT study that found median treatment durations were similar across the second‐line treatments.^18^ We also found no evidence of differences in time to second‐line treatment cessation across the three second‐line treatments (see Table A11).

We predicted counterfactual outcomes for each person for each treatment by combining the individual's own data on baseline characteristics and risk factors (Table A12) with treatment effects from the literature to predict risk factors and complications over the 7.6 years of follow‐up.

We calculated mean predicted values across all patients for up to 7.6 years, but since most people (84%) were censored by year 7, we reported results at 5‐year follow‐up (Table A3). We compared levels of risk factors and probabilities of complications across the second‐line treatments. The risk factors in United States (US) units were: HbA1c (%), BMI (kg/m^2^), SBP (mm Hg), DBP (mm Hg), HDL (mg/dL), LDL (mg/dL), total cholesterol (mg/dL), non‐fasting triglycerides (mg/dL), and eGFR (mL/min/1.73 m^2^). For hypoglycaemia, MI, unstable angina, and stroke we recognised that individuals could experience more than one event over follow‐up (Appendix Stage 1.2: Data S1). We calculated the between‐treatment differences in these mean predictions together with 95% CI, overall and stratified by baseline CVD status. RAPIDS was implemented in R 4.3.1.^30^

## 3 RESULTS

### 3.1 Study population and treatment pathways (stage 1)

**FIGURE 2:**
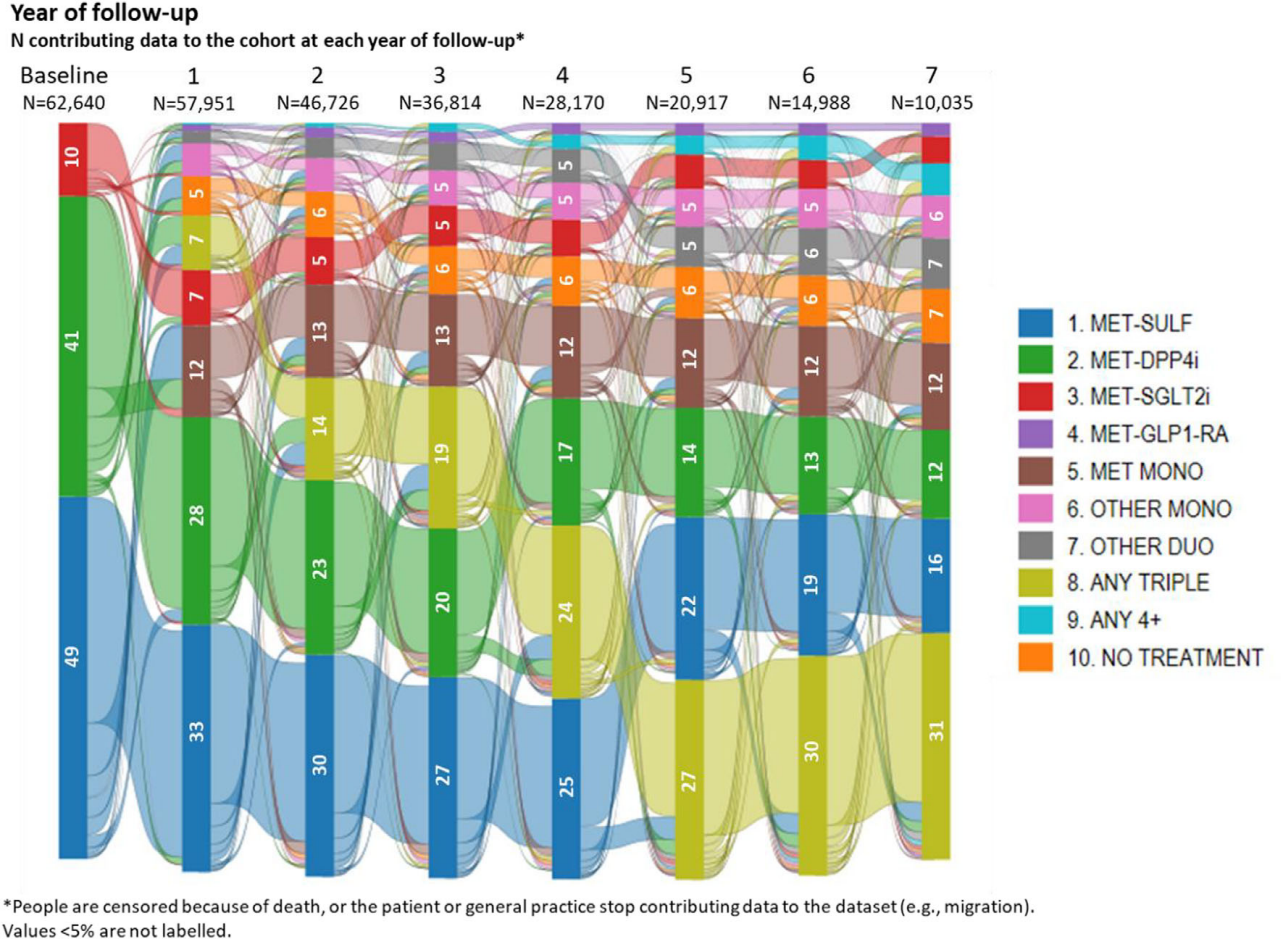
Sankey plot describing the changing proportion of people in the RAPIDS cohort prescribed particular antidiabetic therapies over 7 years follow‐up.

The baseline characteristics are presented in Table 1 and Table A12. Of the patients included, 30 704 (49.0%) were prescribed second‐line treatment with SU, 25539 (40.8%) DPP4i, and 6397 (10.2%) SGLT2i (Table A12). At 1‐year follow‐up, 73% of people were still prescribed second‐line treatment, with 33% metformin‐SU, 28% metformin‐DPP4i, and 7% metformin‐SGLT2i (Figure 2, Tables A13 and A14). For those no longer prescribed dual therapies at 1 year, the most common options were metformin monotherapy (12%) or triple therapy (7%). At the end of follow‐up, the proportions prescribed dual therapies were 16% for metformin‐SU, 12% for metformin‐DPP4i, and 4% for metformin‐SGLT2i with 31% prescribed triple therapy (Tables A13 and A14).

### 3.2 Predictions following the alternative second‐line treatments (stage 4)

#### 3.2.1 Risk factors

Figure A3 presents the predicted mean (95% CI) absolute levels for HbA1c, eGFR, BMI, and SBP, and Figure A4 presents the corresponding results for HDL, LDL, total cholesterol, triglycerides, and DBP. Table A15 summarises the predicted absolute levels for all risk factors. Figures A5 and A6, Table A16 provide the corresponding mean differences in the predicted values of these risk factors for pairwise treatment comparisons of the three alternative second‐line treatments.

Initiating second‐line treatment with SGLT2i was predicted to lead to lower mean HbA1c compared with SU and DPP4i, and lower mean BMI compared with SU across the follow‐up period (Figure A3). By 5 years, SGLT2i lowered mean HbA1c (%) compared to SU by 0.13 (95% CI: −0.14, −0.12), and by 0.40 versus DPP4i (95% CI: −0.41, −0.38). Compared to SU, SGLT2i and DPP4i lowered mean BMI at 5 years (difference of −0.49 kg/m^2^, 95% CI: −0.51, −0.47 for SGLT2i and −0.47, 95% CI: −0.50, −0.45 for DPP4i). By 5 years, those prescribed SGLT2i had higher mean eGFR than those prescribed DPP4i (0.19, 95% CI: 0.05, 0.32), but lower eGFR than those prescribed SU, with a mean difference of −0.09, 95% CI: −0.23, 0.04 (Figure A3, Table A16).

#### 3.2.2 Complications

Figure A7 summarises the absolute predicted probabilities of ESKD, MI, LEA, and HF following each second‐line treatment. Figure A8 presents the corresponding results for hypoglycaemic events, unstable angina, stroke, diabetic eye disease, and all‐cause death. Table A17 summarises the predicted probabilities for all the complications considered.

**FIGURE 3:**
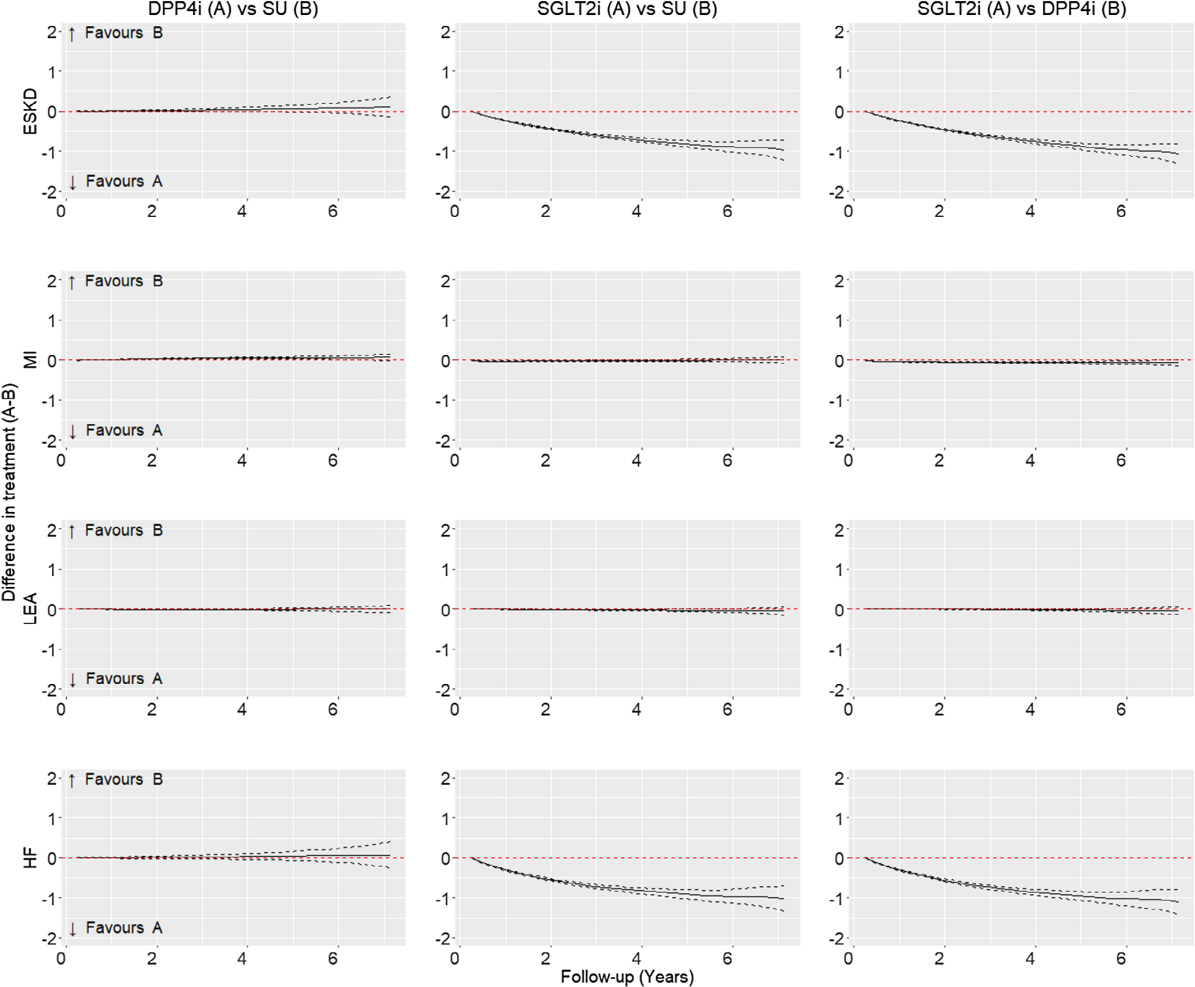
Figure showing the mean difference in the predicted probability (solid black line) for ESKD, MI, lower extremity amputation and HF, compared across counterfactual second‐line oral antidiabetic treatment scenarios: DPP4i vs. SU; SGLT2i vs. SU; SGLT2i vs. DPP4i. The 95% confidence intervals for the difference in predicted probabilities are also presented (dashed black lines). Available follow‐up at year 1 = 57 951, year 2 = 46 726, year 3 = 36 814, year 4 = 28 170, year 5 = 20 917, year 6 = 14 988, and year 7 = 10 035.

**FIGURE 4:**
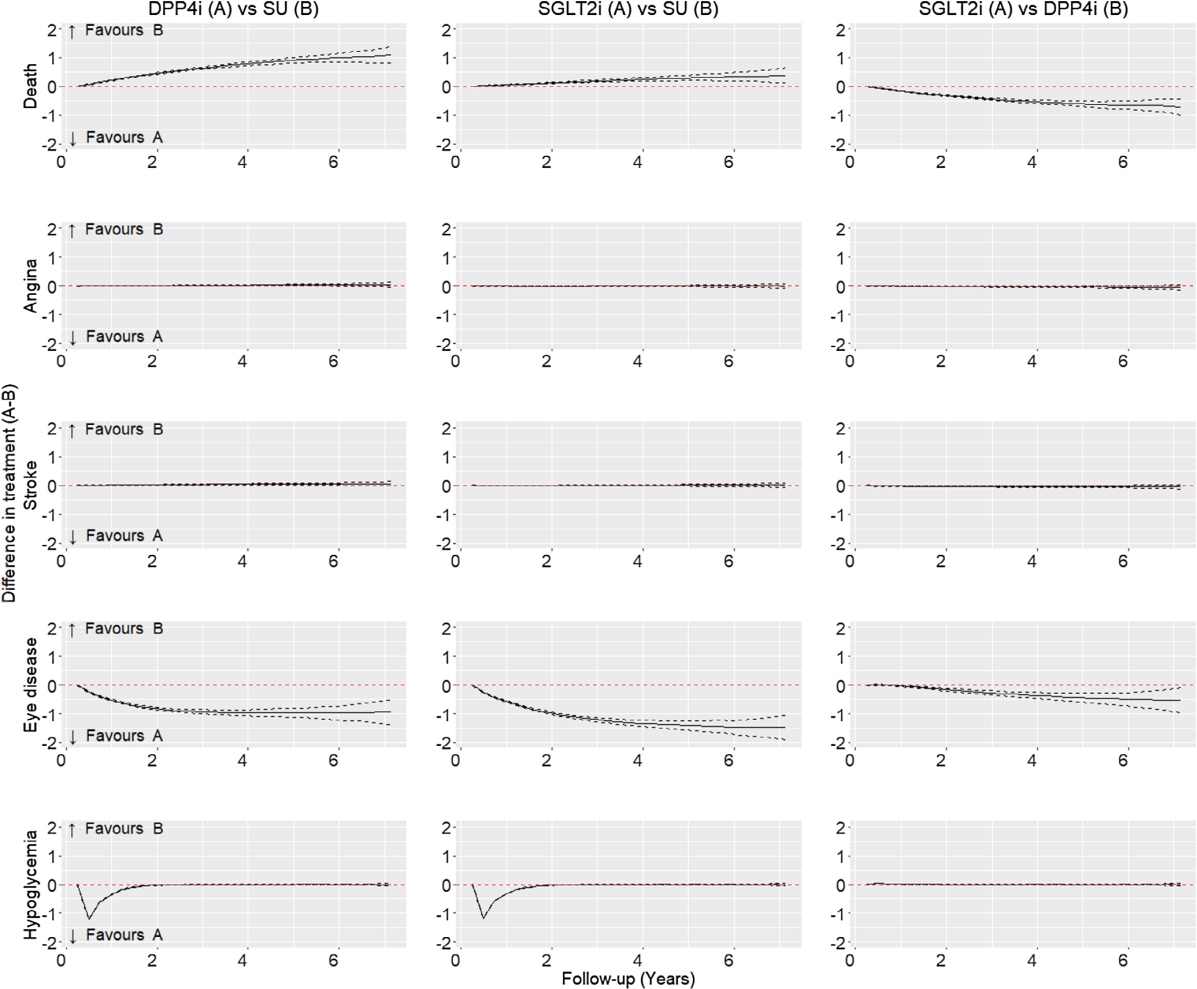
Figure showing the mean difference in the predicted probability (solid black line) for all‐cause death, angina, stroke, eye disease and hypoglycemia, compared across counterfactual second‐line oral antidiabetic treatment scenarios: DPP4i vs. SU; SGLT2i vs. SU; SGLT2i vs. DPP4i. The 95% confidence intervals for the difference in predicted probabilities are also presented (dashed black lines). Available follow‐up at year 1 = 57 951, year 2 = 46 726, year 3 = 36 814, year 4 = 28 170, year 5 = 20 917, year 6 = 14 988, and year 7 = 10 035.

Figure 3 presents mean differences in predicted probabilities of ESKD, MI, LEA, and HF. Figure 4 presents mean differences for hypoglycaemic events, angina, stroke, eye disease, and all‐cause death.

Following second‐line treatment with SGLT2i, the predicted probability of ESKD by 5 years was 3.20% (95% CI: 3.13, 3.27) (Figure 3). The difference (reduction) in the mean probability of ESKD by 5 years for SGLT2i versus SU was −0.81% (−0.89, −0.73) and for SGLT2i versus DPP4i was −0.87% (−0.95, −0.79). The corresponding mean differences (reductions) in the predicted probabilities of HF for SGLT2i versus SU were −0.90% (−1.01, −0.80), and for SGLT2i versus DPP4i were −0.95 (−1.06, −0.84). The mean predicted probability of MI within 5 years following SGLT2i initiation was 0.52% (0.50, 0.54), which was similar to SU, with a mean (95% CI) difference of −0.01 (−0.03, 0.02), and lower than DPP4i, with a mean difference of −0.06 (−0.09, −0.03). The mean probability of LEA following SGLT2i was 0.63% (0.57, 0.69) which was a mean difference (reduction) compared to SU of −0.03 (−0.06, −0.01), and of −0.03 (−0.05, 0.00) versus DPP4i.

The mean probability of diabetic eye disease was 23.00% (22.57, 23.42) for SGLT2i, with a mean difference (reduction) compared to SU of −1.41% (−1.57, −1.26) and compared to DPP4i of −0.44% (−0.59, −0.29). The predicted incidence of hypoglycaemic events increased markedly in the year following second‐line treatment with SU versus either of the alternatives, but did not increase over subsequent follow‐up periods, when the majority of patients switched to alternative treatments. The predicted probabilities of angina, stroke, and all‐cause death were low following all three second‐line treatments (Figure A8, Table A17). The mean differences in the predicted probability of all‐cause death within 5 years following second‐line treatment with SGLT2i were 0.29% (0.20–0.38) (versus SU) and −0.61% (−0.70 to 0.51) versus DPP4i (Figure 4).

In Figures A9–A12, we present the predicted effects of the alternative second‐line treatments on risk factors and complications according to pre‐existing CVD status. We did not find evidence that pre‐existing CVD status modified the impact of second‐line treatment on levels of any of the risk factors (Figures A9 and A10), nor did the probability of complications differ according to pre‐existing CVD status (Figures A11 and A12).

## 4 DISCUSSION

This study finds that initiating second‐line treatment with SGLT2i in routine practice for people with T2DM can reduce the probability of diabetes complications within 5 years compared to SU or DPP4i. The predicted probabilities of HF, ESKD, and diabetic eye disease were all lower following second‐line treatment with SGLT2i versus either SU or DPP4i. The probabilities of MI and LEA at 5 years were similar across the three second‐line treatments.

Initiating second‐line treatment with SGLT2i rather than DPP4i or SU reduced the predicted probability of ESKD and the decline in eGFR (versus DPP4i). These results were in accordance with meta‐analyses, and placebo‐controlled RCTs that reported lower major adverse kidney events among those randomised to SGLT2i.^10^, ^11^, ^12^, ^31^, ^32^, ^33^, ^34^ The finding that SGLT2i led to lower incidence of HF hospitalisation is also consistent with previous meta‐analyses of RCTs.^10^, ^12^ Our findings of no difference in the predicted probability of MI among the alternative second‐line treatment aligns with some published RCTs.^35^, ^36^ Other RCTs^6^, ^7^ which reported fewer CVD events following SGLT2i versus ‘placebo’ are difficult to interpret as the definitions of the ‘placebo arm’ differed within and across the studies according to the specifics of the RCT protocol and the preferred second‐line drugs at that time.

Our study adds to evidence from cardiovascular outcomes trials (CVOT) in predicting the risk of complications following alternative second‐line treatments for a broader population of patients with T2DM who have second‐line treatment in routine practice. These patients were on average aged 60 years had only been diagnosed with T2DM for 5 years, and only 14% had previous CVD. By contrast, the patients included in the CVOT tend to be older, had been diagnosed with T2DM for longer, and tended to have previous CVD or kidney disease. For example, across the six RCTs included in the systematic review by Kunutusor et al., the average age was 65, the T2DM duration was 13 years, and most of the CVOT included patients at high risk of renal complications or CVD.^12^ By using baseline characteristics and risk factor profiles from a general population presenting for second‐line treatment in routine practice, we were able to predict the relative impact of second‐line treatment with SGLT2i versus alternatives on the incidence of diabetes complications in the ‘real world’.

The study adds to limited evidence about the effect of SGLT2i on major microvascular complications including diabetic eye disease (retinopathy and/or blindness) and LEA. Meta‐analyses have shown that SGLT2i have nephroprotective properties,^12^ supported by evidence of reduced risk of kidney events, but not of lower rates of diabetic eye disease. While it is well established that long‐term good glycaemic control can reduce the eventual risk of diabetic retinopathy development and progression, it is encouraging that the RAPIDS model predicts further reductions in the incidence of eye disease within 5 years following second‐line treatment with SGLT2i versus DPP4i or SU.

There has been caution in using SGLT2i after the Food and Drug Administration (FDA) in the USA issued Drug Safety Communication that canagliflozin causes increased risk of leg and foot amputations based on two RCTs comparing canagliflozin to placebo.^37^ However, our findings are similar to those from a systematic review of RCTs that reported SGLT2i was not associated with foot amputations.^38^ A meta‐analysis of observational studies that directly compared the risk of lower limb amputations also found that the risk of lower limb amputations was similar for patients who used SGLT2i and DPP4i, and that physicians should not fear increased risk of lower limb amputation for patients prescribed SGLT2i in routine clinical practice.^39^

Clinical guidelines recommend second‐line treatment with SGLT2i for those with pre‐existing CVD, high risk of CVD, heart failure, or kidney disease.^3^, ^4^ We did not find any evidence of additional improvement in outcomes following second‐line treatment with SGLT2i versus DPP4i or SU according to CVD status. A published meta‐analysis of RCTs also did not find evidence that the effect of SGLT2i (vs placebo) on major adverse cardiovascular events differed according to pre‐existing CVD status.^12^

The paper predicts risk factors and complications from the real‐world patterns of treatment use, not just of second‐line treatments but from subsequent treatment changes. We recognise that after second‐line treatment people may take triple therapies which may include insulin or GLP1‐RA, but also that patients may switch to monotherapy. So we were able to use these real‐world treatment patterns to provide realistic estimates of the long‐term effectiveness of initiating SGLT2i at second‐line. These subsequent treatment choices, which were similar to those observed in other settings,^40^ were observed over a longer follow‐up (7 years) than recent CVOT including those with novel therapies and included a larger cohort of patients representative of those presenting for second‐line treatment in routine practice.^6^, ^7^, ^31^, ^32^, ^41^

The model estimates the impact of initiating different second‐line treatments across a full range of long‐term events of direct interest to patients, both via clinical measures such as HbA1c and BMI (indirect effects), but also as direct effects. The model provided more holistic estimates on microvascular and macrovascular complications as well as risk factors of the alternative second‐line treatments than are available from RCTs. The RAPIDS‐UK model followed the published updates to the RAPIDS model (2.0)^17^ by using treatment effectiveness parameters from meta‐analyses of published RCTs. The RAPIDS‐UK model was subject to a careful calibration assessment, and minor changes were made to recalibrate the model to the population of people with T2DM presenting for second‐line treatment in England. The recalibrated model gave accurate predictions for all long‐term complications within 5 years versus those in the observed data.

The paper has several limitations. First, this paper does not consider second‐line treatment with GLP1‐RA. While use of this drug class has increased world‐wide in recent years, GLP1‐RA for the specific indication of glycaemic control are often only recommended as third‐ or subsequent lines of glucose‐lowering therapy, for example by NICE in England,^3^ and their use as second‐line treatment in most countries is low.^42^ As data emerges on the use of GLP1‐RA and other peptides, the RAPIDS model can consider the comparative effectiveness of these therapies at different places across the treatment pathway (first‐, second‐, and third‐ line). This would help decision‐makers decide when additional costs of new therapies may be justified by important outcome gains. This current paper does not consider the optimum place in the treatment pathway for drugs such as SGLT2i and GLP1‐RA.

Second, the model does not consider the overall relative value of preventing the different events, nor the total costs offset. For the current comparison, an extension to include endpoints such as Quality Adjusted Life Years and total costs is unlikely to yield a new conclusion. On the outcomes side, the current version of the model finds that initiating second‐line treatment with metformin‐SGLT2i leads to similar or better outcomes than its comparators. On the costs side, although the current list price of SGLT2i is higher than for DPP4i and SU, it is anticipated that once the current licence for exclusivity ends, drug prices will be fairly similar across the alternatives. Hence, as the model predicts that metformin‐SGLT2i reduces the incidence of high‐cost events such as ESKD, it can be anticipated that they would be relatively cost‐effective compared to other therapies.

Third, the model predicted a small increase (0.29%) in the probability of all‐cause death following second‐line treatment with SGLT2i versus SU. This finding should be regarded with caution as it is contrary to the extant literature which finds that all‐cause death is similar or lower following SGLT2i compared to placebo,^12^ and that there is no obvious mechanism as to why all‐cause death is higher following second‐line treatment with SGLT2i versus SU. We believe this finding may be a manifestation of the uncertainty with which all‐cause mortality is predicted in this cohort, and further research to resolve it would be worthwhile. Finally, RAPIDS is a computationally intensive model, and implementing it took several months. This reduced our ability to implement alternative internal validation methods or run multiple sensitivity analyses.

## 5 CONCLUSION

In conclusion, for people with T2DM, the RAPIDS‐UK model predicts a lower proportion of people with cardiorenal complication at 5 years after second‐line glucose‐lowering treatment in routine practice with SGLT2i compared with SU or DPP4i added to metformin monotherapy.

## FUNDING INFORMATION

This work was funded by the National Institute for Health and Care Research (NIHR) grant number NIHR128490. KK is supported by the NIHR Applied Research Collaboration East Midlands (ARC EM), NIHR Global Research Centre for Multiple Long Term Conditions, NIHR Cross NIHR Collaboration for Multiple Long Term Conditions, and the NIHR Leicester Biomedical Research Centre (BRC). AA is supported by the NIHR Oxford Biomedical Research Centre (BRC). The funder had no role in considering the study design or in the collection, analysis, interpretation of data, writing of the report, or decision to submit the article for publication.

## CONFLICT OF INTEREST STATEMENT

Kamlesh Khunti has acted as a consultant for, been a speaker for, has received a grant for investigator initiated studies from: AstraZeneca, Bayer, Novo Nordisk, Sanofi‐Aventis, Servier, Eli Lilly, Boehringer‐Ingelheim, Oramed Pharmaceuticals, Pfizer, Roche, Daiichi‐Sankyo, Applied Therapeutics, Embecta, and Nestle Health Science. Amanda I. Adler has received income from Novo‐Nordisk. Andrew H. Briggs is an ecomic advisor to the DIRECT trial. Anirban Basu has served as a consultant to the following pharmaceutical companies within the last three years: Astra Zeneca, Boeringer Ingelheim, Daiichi‐Sankyo, Eli Lilly, Gilead, Idorsia, Novo Nordisk. None of the other authors have any conflicts of interest to declare.

## PEER REVIEW

The peer review history for this article is available at https://www.webofscience.com/api/gateway/wos/peer-review/10.1111/dom.16447.

## Supporting information


**Data S1.** Appendix.

## Data Availability

We cannot share CPRD‐linked data used in this study. However, CPRD data of the form used in this paper are available after protocol approval via CPRD's Research Data Governance process. Further details can be found here: https://www.cprd.com/data-access.
