# Supplementary material for: Long‐term outcomes following alternative second‐line oral glucose‐lowering treatments: Results from the real‐world progression in type 2 diabetes mellitus United Kingdom (RAPIDS‐UK) model
Source: Diabetes Obes Metab. 2025 May 21;27(8):4181–91. doi: 10.1111/dom.16447 (PMC12232352; doi:10.1111/dom.16447)
Supplement: Supplementary file 1 — Data S1. Appendix. [file DOM-27-4181-s001.docx]

**Appendices**

Long-term outcomes following alternative second-line oral glucose-lowering treatments: results from the Real-World Progression in type 2 diabetes mellitus United Kingdom (RAPIDS-UK) model

**Contents**

[Stage 1: Definition of study population and treatment pathways 6](#_Toc194393489)

[**Table A1:** Details of inclusion and exclusion criteria for the study population for the RAPIDS model 7](#_Toc194393490)

[1.1. Handling missing data for risk factors 8](#_Toc194393491)

[1.2. Variables and study populations available for the RAPIDS-UK versus previous RAPIDS (US) models 9](#_Toc194393492)

[**Table A2:** Comparison of study populations used for RAPIDS-UK versus previous versions 9](#_Toc194393493)

[**Table A3:** Sample size for eligible CPRD cohort at different follow-up time points in the RAPIDS model* 10](#_Toc194393494)

[**Table A4:** Definitions of complications in the UK primary and secondary care data populating the RAPIDS model. 11](#_Toc194393495)

[**Table A5:** Definition of CVD history prior to second-line treatment initiation 12](#_Toc194393496)

[Stage 2: Implementing the RAPIDS model (see also Basu et al, 2024^3^) 13](#_Toc194393497)

[**Table A6:** Effects of DPP4i and SGLT2i monotherapies, and metformin-DPP4i and metformin-SGLT2i versus metformin monotherapy (unless otherwise stated) from the literature (over 52 weeks) 14](#_Toc194393498)

[**Table A7:** RAPIDS 90-day quarter treatment effectiveness input for second-line treatment added to metformin versus no treatment 16](#_Toc194393499)

[**Table A8:** Translated quarterly effects of DPP4i and SGLT2i monotherapies, and metformin-DPP4i, and metformin-SGLT2i versus no treatment on various outcomes for the RAPIDS risk engine. 17](#_Toc194393500)

[**Table A9:** RAPIDS 90-day quarter treatment effectiveness input for alternative second-line treatments added to metformin versus no treatment. 19](#_Toc194393501)

[Stage 3 Model calibration 20](#_Toc194393502)

[**3.1 Methods** 20](#_Toc194393503)

[**Table A10:** Baseline characteristics of the CPRD cohort, overall and stratified by training and validation split 21](#_Toc194393504)

[**3.2 Calibration results** 23](#_Toc194393505)

[Stage 4: Comparison of alternative second-line treatments 26](#_Toc194393506)

[**Table A11:** Baseline characteristics of patients with type 2 diabetes mellitus 2011-2020 from the CPRD eligible cohort, stratified by 2^nd^ line treatment initiated, added to metformin 27](#_Toc194393507)

[**Table A12:** Assessment of the time to second-line glucose-lowering treatment cessation across the alternative second-line treatments (N=54,033) 30](#_Toc194393508)

[**Table A13:** Observed glucose-lowering treatment courses (proportion of people prescribed) over each year of follow-up included in the RAPIDS model categorised according to the Sankey plot (main text Figure 1) 31](#_Toc194393509)

[**Table A14:** Observed glucose-lowering treatment courses (proportion of people prescribed) over each year of follow-up in the RAPIDS model 32](#_Toc194393510)

[**Table A15:** Five-year estimates of the predicted mean values of the risk factors (biomarkers) under each counterfactual pathway 37](#_Toc194393511)

[**Table A16:** Five-year estimates of the mean difference in predicted values of the risk factors (biomarkers) across counterfactual second-line oral antidiabetic treatment scenarios 40](#_Toc194393512)

[**Table A17:** Five-year estimates of the mean predicted probabilities of complications under each counterfactual pathway 43](#_Toc194393513)

[**Table A18:** Five-year estimates of the mean difference in predicted mean values of the complications across counterfactual second-line oral antidiabetic treatment scenarios. 44](#_Toc194393514)

[References 49](#_Toc194393515)

**Table of Appendix Tables**

[**Table A1:** Details of inclusion and exclusion criteria for the study population for the RAPIDS model 7](#_Toc194393516)

[**Table A2:** Comparison of study populations used for RAPIDS-UK versus previous versions 9](#_Toc194393517)

[**Table A3:** Sample size for eligible CPRD cohort at different follow-up time points in the RAPIDS model* 10](#_Toc194393518)

[**Table A4:** Definitions of complications in the UK primary and secondary care data populating the RAPIDS model. 11](#_Toc194393519)

[**Table A5:** Definition of CVD history prior to second-line treatment initiation 12](#_Toc194393520)

[**Table A6:** Effects of DPP4i and SGLT2i monotherapies, and metformin-DPP4i and metformin-SGLT2i versus metformin monotherapy (unless otherwise stated) from the literature (over 52 weeks) 14](#_Toc194393521)

[**Table A7:** RAPIDS 90-day quarter treatment effectiveness input for second-line treatment added to metformin versus no treatment 16](#_Toc194393522)

[**Table A8:** Translated quarterly effects of DPP4i and SGLT2i monotherapies, and metformin-DPP4i, and metformin-SGLT2i versus no treatment on various outcomes for the RAPIDS risk engine. 17](#_Toc194393523)

[**Table A9:** RAPIDS 90-day quarter treatment effectiveness input for alternative second-line treatments added to metformin versus no treatment. 19](#_Toc194393524)

[**Table A10:** Baseline characteristics of the CPRD cohort, overall and stratified by training and validation split 21](#_Toc194393525)

[**Table A11:** Baseline characteristics of patients with type 2 diabetes mellitus 2011-2020 from the CPRD eligible cohort, stratified by 2^nd^ line treatment initiated, added to metformin 27](#_Toc194393526)

[**Table A12:** Assessment of the time to second-line glucose-lowering treatment cessation across the alternative second-line treatments (N=54,033) 30](#_Toc194393527)

[**Table A13:** Observed glucose-lowering treatment courses (proportion of people prescribed) over each year of follow-up included in the RAPIDS model categorised according to the Sankey plot (main text Figure 1) 31](#_Toc194393528)

[**Table A14:** Observed glucose-lowering treatment courses (proportion of people prescribed) over each year of follow-up in the RAPIDS model 32](#_Toc194393529)

[**Table A15:** Five-year estimates of the predicted mean values of the risk factors (biomarkers) under each counterfactual pathway 37](#_Toc194393530)

[**Table A16:** Five-year estimates of the mean difference in predicted values of the risk factors (biomarkers) across counterfactual second-line oral antidiabetic treatment scenarios 40](#_Toc194393531)

[**Table A17:** Five-year estimates of the mean predicted probabilities of complications under each counterfactual pathway 43](#_Toc194393532)

[**Table A18:** Five-year estimates of the mean difference in predicted mean values of the complications across counterfactual second-line oral antidiabetic treatment scenarios. 44](#_Toc194393533)

**Table of Appendix Figures**

[**Figure A1:** Predicted versus observed risk factors (biomarker values) and complications (events) for people who initiate second-line oral glucose-lowering treatment with one of SU, DPP4i, or SGLT2i, added to metformin after applying the US RAPIDS 2.0 calibration model 24](#_Toc194393534)

[**Figure A2:** Predicted versus observed risk factors (biomarker values) and complications (events) for people who initiate second-line oral glucose-lowering treatment with one of SU, DPP4i, or SGLT2i, added to metformin after applying the calibration update to the UK population 25](#_Toc194393535)

[**Figure A3:** Figure showing the predicted mean absolute values for HbA1c (%), eGFR (mL/min/1.73m2), SBP (mm Hg), and BMI (kg/m2) from the RAPIDS-UK model. The solid black line shows the predicted mean values and the dotted lines represent 95% confidence intervals. A range in HbA1c of 7.5-8.5% is equivalent to 58-69mmol/mol. 35](#_Toc194393536)

[**Figure A4:** Figure showing the predicted mean absolute values for HDL (mg/dL), LDL (mg/dL), total cholesterol (mg/dL), triglycerides (mg/dL), and DBP (mm Hg) in the RAPIDS model. The solid black line shows the predicted mean values and the dotted lines represent 95% confidence intervals. 36](#_Toc194393537)

[**Figure A5:** Mean difference in the predicted mean levels of for HbA1c (%), eGFR (mL/min/1.73m2), SBP (mm Hg), and BMI (kg/m2) (solid black lines) across counterfactual second-line oral antidiabetic treatment scenarios: DPP4i vs. SU; SGLT2i vs. SU; SGLT2i vs. DPP4i. The 95% confidence intervals for the difference in predicted probabilities are also presented (dashed black lines). A change in HbA1c of ±0.2% is equivalent to ±2.2mmol/mol. 38](#_Toc194393538)

[**Figure A6:** Figure showing the mean difference in the predicted mean values for HDL (mg/dL), LDL (mg/dL), total cholesterol (mg/dL), triglycerides (mg/dL), and DBP (mm Hg), compared across counterfactual second-line oral antidiabetic treatment scenarios. The solid black lines show the mean difference and the dotted lines the 95% confidence interval. 39](#_Toc194393539)

[**Figure A7:** Figure showing the predicted mean absolute probabilities for ESRD, MI, LE amputation and HF from the RAPIDS model. The solid black line shows the predicted mean values and the dotted lines represent 95% confidence intervals. 41](#_Toc194393540)

[**Figure A8:** Figure showing the predicted mean probabilities for all-cause death, angina, stroke, diabetic eye disease, and hypoglycaemia in the RAPIDS model. The solid black line shows the predicted mean values and the dotted lines represent 95% confidence intervals. 42](#_Toc194393541)

[**Figure A9:** Figure showing the mean difference in the predicted mean values for HbA1c (%), eGFR (mL/min/1.73m2), SBP (mm Hg), and BMI (kg/m2), compared across counterfactual second-line oral antidiabetic treatment scenarios by CVD status. The solid lines show the mean difference and the dotted lines the 95% confidence interval. A change in HbA1c of ±0.2% is equivalent to ±2.2mmol/mol. 45](#_Toc194393542)

[**Figure A10:** Figure showing the mean difference in the predicted mean values for HDL (mg/dL), LDL (mg/dL), total cholesterol (mg/dL), triglycerides (mg/dL), and DBP (mm Hg), compared across counterfactual second-line oral antidiabetic treatment scenarios by CVD status. The solid lines show the mean difference and the dotted lines the 95% confidence interval. 46](#_Toc194393543)

[**Figure A11:** Figure showing the mean difference in the predicted probability for ESKD, MI, LE amputation, and HF, compared across counterfactual second-line oral antidiabetic treatment scenarios by CVD status. The solid lines show the mean difference and the dotted lines the 95% confidence interval. 47](#_Toc194393544)

[**Figure A12:** Figure showing the mean difference in the predicted probability for all-cause death, angina, stroke, diabetic eye disease, and hypoglycaemia, compared across counterfactual second-line oral antidiabetic treatment scenarios by CVD status. The solid lines show the mean difference and the dotted lines the 95% confidence interval. 48](#_Toc194393545)

# **Stage 1: Definition of study population and treatment pathways**

We designed PERMIT to assess the short- and long-term comparative effectiveness of SGLT2i versus DPP4i versus SU as add-ons to metformin monotherapy for people with type 2 diabetes in routine clinical practice. The cohort eligibility criteria (Supplementary table 1) required that people were aged 18 years or older, diagnosed with type 2 diabetes, registered with a GP in England, and intensified treatment from first- to second-line oral antidiabetic treatment between 1 January 2015 to 31 December 2020 with a first-ever prescription of SU, DPP4i or SGLT2i, added to metformin.

For the RAPIDS-UK study, we extended the study period to 1 January 2011 to 31 December 2020. Information may no longer be available i.e., censored if the GP no longer contributes data to CPRD, or that individual no longer attends the GP practice or due to death. The sample size of patients across various follow-up time points is presented in Table 2**.** Eligible patients had at least one prescription for metformin monotherapy within 60 days *prior* to the first prescription for second-line treatment, to increase the probability of the continuous use of metformin monotherapy prior to intensification. We excluded women with a record of pregnancy within 12 months prior to second-line treatment initiation, people whose last recorded eGFR was less than 30mL/min/1.73m2, people whose GPs had not consented to the required linkage of HES data, and those who were not prescribed metformin on the same day as, or within 60 days *after,* initiating second-line treatment.^1^

Marital and veteran status are not collected information in CPRD or HES, but RAPIDS requires this information. Each patient was set as, “not a veteran” and with a 50% possibility of being married.2 The “South Asian” and “Mixed/Other” ethnicity categories in CPRD did not easily fit into the “Hispanic”, “non-Hispanic White” and “non-Hispanic African American” categories required for RAPIDS and we therefore included them in the reference category.

### **Table A1:** Details of inclusion and exclusion criteria for the study population for the RAPIDS model

| **Inclusion criteria** | **Exclusion criteria** |
| --- | --- |
| - Aged ≥18 years - T2DM diagnosis code, to avoid including people prescribed antidiabetic drugs for other indications (e.g., polycystic ovarian syndrome). - Prescribed metformin monotherapy as first-line oral antidiabetic treatment, on the same day or following a T2DM diagnosis. - Registered with a GP in England with acceptable data standards flag by CPRD (to help ensure adequate data availability). - Registered with GP for ≥1 year prior to first metformin prescription (to help ensure adequate baseline data availability and reduce recording of past events as incident). - Initiated SU, DPP4i, or SGLT2i between 1 January 2011 to 31 December 2020 (the study period). - At least 1 metformin prescription within 60 days prior to new second-line drug, and at least one metformin prescription on the same day or within 60 days after new second-line drug, to ensure the person is adding on to metformin and not switching. - Linked to HES/ONS/IMD data (to help ensure outcomes are captured). - Complete data after interpolation for all variables included in the RAPIDS model. | - Prescribed >1 non-metformin antidiabetic drugs on the date of second-line treatment initiation (beyond study scope). - Initiates second-line oral antidiabetic treatment with drug class other than SU, DPP4i, or SGLT2i (beyond study scope). - Latest eGFR recorded by the GP prior to second-line oral antidiabetic treatment initiation is <30mL/min/1.73m^2^ (since at the time of data-collection most GPs would not have prescribed metformin for people with eGFR<30mL/min/1.73m^2^; the results from the DAPA-CKD trial (which did randomise people with eGFR less than 30mL/min/1.73m^2^) were only available towards the very end of the study period and are unlikely to have informed decisions taken in primary care). - Women who have a record of pregnancy in primary care within 1 year prior to second-line antidiabetic treatment initiation (since guidelines are different for this group). |

## Handling missing data for risk factors

A patient must have two fully observed quarters to meet inclusion criteria for the RAPIDS model. The first quarter is defined as the first period for a patient where all risk factors (biomarkers) are present. Biomarker values can be present in a quarter from either (i) being measured during the quarter or (ii) imputed using linear interpolation. We will allow for historical biomarker values recorded up to 10 years prior to second-line treatment initiation to be used in the linear interpolation to impute missing biomarker data during the follow-up time of interest (each quarter following second-line treatment initiation). We will not extrapolate missing values prior to the first observed value or beyond the last observed value for all biomarkers.

Values for a biomarker must be present at some point in a patient’s history to use linear interpolation. A biomarker, *b*, can have a missing value at quarter, *q*, imputed using:

$$b_{q}= b_{(q-1)}+(q-\left( q-1 \right))\frac{b_{(q+1)}-b_{(q-1)}}{\left( q+1 \right)-(q-1)}$$

Where $(q+1)$ represents the time period for the next observable biomarker value after quarter $q$ and $(q-1)$ represents the time period for the previous observable biomarker before quarter $q$.

Once a patient’s first two quarters were established, they were run through the RAPIDS model. The RAPIDS model estimated predicted values within each quarter, and variables such as age or history of a cardiovascular event were updated at the start of each new quarter.

For each 90-day quarter, RAPIDS required only one observed value for each biomarker and treatment. Within a 90-day period, if the person had more than one clinical measure we took the mean value, and if they did not have the measure required, we imputed that measure using linear interpolation.

## 1.2. Variables and study populations available for the RAPIDS-UK versus previous RAPIDS (US) models

RAPIDS was originally developed on the Veterans Affairs (VA) dataset and was then updated to a general US database (RAPIDS 2.0).3 The UK CPRD data runs from 2011-2020 in adults ≥18 years of age with T2DM. Variables such as marital status and veteran status were not present in CPRD, and so it was assumed that 50% were married, and none were veterans. All other requisite variables were available (see **Table A2**).

### **Table A2:** Comparison of study populations used for RAPIDS-UK versus previous versions

| **Characteristics** | **Veteran Affair data (RAPIDS 1.0)** | **US General Electronic Medical Records (RAPIDS 2.0)** | **RAPIDS-UK** |
| --- | --- | --- | --- |
| **Purpose** | Development of RAPIDS | Generalisability to US population | External validation application to UK T2DM patients |
| **N (training + validation)** | 307k + 105k + 105k | 25k + 263k | 20k + 42.5k |
| **Time period** | 2003-2010 | 2008-2021 | 2011-2020 |
| **Time period** | 2003-2010 | 2008-2021 | 2011-2020 |
| **Age (years)** | ≥40 | 30-85 | ≥18 |
| **Ethnicity** | Non-Hispanic black  Non-Hispanic white  Hispanic | Non-Hispanic black  Non-Hispanic white  Hispanic | White  South Asian  Black  Mixed/Other |
| **Marital status** | Married  Never married | Not available in data | Not available in data |
| **Veteran status** | Yes  No | Not available in data | Not available in data |

### **Table A3:** Sample size for eligible CPRD cohort at different follow-up time points in the RAPIDS model*

| **Year** | **Patient count, n (%)** |
| --- | --- |
| 0 | 62,640 (100.0) |
| 1 | 57,951 (92.5) |
| 2 | 46,726 (74.6) |
| 3 | 36,814 (58.8) |
| 4 | 28,170 (45.0) |
| 5 | 20,917 (33.4) |
| 6 | 14,988 (23.9) |
| 7 | 10,035 (16.0) |

*Note that the declining number of patients available reflects censoring study due to the end of the study data period (31 December 2020), or if the GP no longer contributed data to CPRD, if the individual no longer attended the GP practice, or due to death. The RAPIDs model assumes that this censoring is ‘at random’ i.e. after conditioning on the variables included within the RAPIDS model.^2^

### **Table A4:** Definitions of complications in the UK primary and secondary care data populating the RAPIDS model.

| **Outcome** | **Definitions** |
| --- | --- |
| Myocardial infarction | Primary care: diagnosis code (Read or Snomed) for myocardial infarction. Secondary care: diagnosis code (ICD-10) for myocardial infarction (first or second diagnostic position in any episode of a spell). |
| Unstable angina | Primary care: diagnosis code (Read or Snomed) for unstable angina.  Secondary care: diagnosis code (ICD-10) for unstable angina (first or second diagnostic position in any episode of a spell). |
| Stroke | Primary care: diagnosis code (Read or Snomed) for stroke.  Secondary care: diagnosis code (ICD-10) for stroke (first or second diagnostic position in any episode of a spell). |
| Heart failure hospitalisation | Secondary care: diagnosis code (ICD-10) for heart failure (first or second diagnostic position in any episode of a spell). |
| Hypogylcaemia | Primary care: diagnosis code (Read or Snomed) for hypoglycaemia or hyperinsulinism.  Secondary care: diagnosis code (ICD-10) for hypoglycaemia. |
| Diabetic eye disease | Primary care: diagnosis code (Read or Snomed) for retinopathy or blindness. |
| Lower limb amputation | Primary care: diagnosis code (Read or Snomed) for lower-limb amputation.  Secondary care: diagnosis code (ICD-10) for limb amputation (first or second diagnostic position in any episode of a spell). |
| End-stage kidney disease (ESKD) | Primary care: diagnosis code (Read or Snomed) for ESKD, dialysis, or kidney transplant.  Secondary care: diagnosis code (ICD-10) in first or second diagnostic position of any episode in a spell, or procedure codes (OPCS) for additional kidney replacement therapy, dialysis, or kidney transplant. |

### **Table A5:** Definition of CVD history prior to second-line treatment initiation

| **Condition part of the CVD history composite variable** | **Definition** |
| --- | --- |
| History of ischaemic heart disease | Primary care: diagnosis code (Read or Snomed) for ischaemic heart disease.  Secondary care: diagnosis code (ICD-10) for ischaemic heart disease (any diagnostic position in any episode of a spell). |
| History of myocardial infarction | Primary care: diagnosis code (Read or Snomed) for myocardial infarction.  Secondary care: diagnosis code (ICD-10) for myocardial infarction (any diagnostic position in any episode of a spell). |
| History of unstable angina | Primary care: diagnosis code (Read or Snomed) for unstable angina.  Secondary care: diagnosis code (ICD-10) for unstable angina (any diagnostic position in any episode of a spell). |
| History of congestive heart failure | Primary care: diagnosis code (Read or Snomed) for congestive heart failure.  Secondary care: diagnosis code (ICD-10) for heart failure (any diagnostic position in any episode of a spell). |
| History of stroke | Primary care: diagnosis code (Read or Snomed) for stroke.  Secondary care: diagnosis (ICD-10) for stroke (any diagnostic position in any episode of a spell). |

# **Stage 2: Implementing the RAPIDS model (see also Basu et al, 2024^3^)**

**2.1 Incorporating estimates of treatment effects from the RAPIDS 2.0 model *(see Basu et al, 2024)***

RAPIDS 2.0 incorporated the monotherapy and combination therapy with metformin effects of DPP4i and SGLT2i. These effects were extracted from trial data and recent meta-analyses (Table A6). These effects were commonly compared with placebo or metformin monotherapy. The effects were converted to be compared to “no treatment” by adding the existing effects of metformin monotherapy versus no therapy estimated from the original RAPIDS model. More specifically, the effects of HbA1c, LDL, Total Cholesterol, and eGFR for DPP4i and SGLT2i were converted to be compared to no treatment by adding the mean effects of metformin monotherapy from the original RAPIDS model (Table A7).

### **Table A6:** Effects of DPP4i and SGLT2i monotherapies, and metformin-DPP4i and metformin-SGLT2i versus metformin monotherapy (unless otherwise stated) from the literature (over 52 weeks)

| **Risk factors & complications** | **Parameter Type** | **Monotherapy** | | **Combo Therapy with metformin** | |
| --- | --- | --- | --- | --- | --- |
|  |  | **DPP4i^6^** | **SGLT2i^7-9^** | **DPP4i^6,10^** | **SGLT2i^7^** |
| BMI (kg/m2) | ARR | 0.478 (0.055) | -0.691 (0.041)^8^ | -0.705 (0.078)^10^ | -0.742 (0.037) |
| Haemoglobin A1c (%) | ARR | 0.430 (0.060) | -0.520 (0.028)^9^ | 0.250 (0.025)^11^ | -0.550 (0.033) |
| High-density lipoprotein (mg/dL) | ARR |  |  |  | 0.050 (0.005) |
| Low-density lipoprotein (mg/dL) | ARR |  |  |  | 0.040 (0.030) |
| Total Cholesterol (mg/dL) | ARR |  |  |  |  |
| Triglyceride (mg/dL) | ARR |  |  |  |  |
| Systolic BP (mm Hg) | ARR |  | -2.460 (0.200)8 |  | -3.620 (0.303) |
| Diastolic BP (mm Hg) | ARR |  | -1.460 (0.183)8 |  | -1.460 (0.183)^8^ |
| EGFR (mL/min/1.73m2) | ARR |  | 0.550 (0.600)12 |  | 0.550 (0.600)^12^ |
| Death | LRR |  |  |  | -0.139 (0.040) |
| Myocardial Infarction | LRR |  |  |  | -0.105 (0.050) |
| Any unstable angina | LRR |  |  |  |  |
| Stroke | LRR |  |  |  | -0.02 (0.066) |
| Any hypoglycaemia | LRR |  | 0.239 (0.094)^9^ | 0.03 (0.219)^6^ | 0.239 (0.094) |
| Congestive heart failure | LRR |  | -0.357 (0.057)^7^ |  | -0.357 (0.057) |
| Lower extremity amputation | LRR |  |  |  |  |
| Advanced eye disease | LRR |  |  |  |  |
| ESRD | LRR |  | -0.478 (0.049)^13,14^ |  | -0.478  (0.049)^13,14^ |

ARR: Absolute risk reduction; LRR: Ln(Relative Risk). Blank cells indicate a lack of evidence which we assume to be a null effect.

Effect on BMI was constructed by dividing the effects on weight (in kgs) by 1.65m^2^

### **Table A7:** RAPIDS 90-day quarter treatment effectiveness input for second-line treatment added to metformin versus no treatment

| **Measure** | **Biomarkers and Long-**  **term outcomes** | **Metformin +** | | |
| --- | --- | --- | --- | --- |
|  |  | **SU** | **DPP4i** | **SGLT2i** |
| Parameter Source | | RAPIDS model | Literature | Literature |
| ARR | BMI (kg/m2) | 0.014 (0.005) | -0.176 (0.020) | -0.185 (0.009) |
|  | HbA1c (%) | -0.151 (0.050) | -0.026 (0.052) | -0.203 (0.022) |
|  | HDL (mg/dL) | -0.210 (0.100) |  | 0.013 (0.001) |
|  | LDL (mg/dL) | -0.872 (0.300) | -0.599 (0.200) | -0.589 (0.200) |
|  | Total Cholesterol (mg/dL) | -0.769 (0.350) | -0.482 (0.200) | -0.482 (0.200) |
|  | Triglycerides, non-fasting  (mg/dL) | 1.500 (0.750) |  |  |
|  | SBP (mm Hg) |  |  | -0.905 (0.076) |
|  | DBP (mm Hg) |  |  | -0.365 (0.046) |
|  | eGFR (mL/min/1.73m2) | -0.483 (0.200) | -0.672 (0.300) | -0.535 (0.335) |
| LRR | Death | -0.003 (0.001) |  | -0.035 (0.010) |
|  | MI |  |  | -0.026 (0.012) |
|  | Unstable Angina |  |  |  |
|  | Stroke |  |  | -0.005 (0.017) |
|  | Hypoglycemia | 0.040 (0.010) | 0.008 (0.055) | 0.060 (0.024) |
|  | CHF |  |  | -0.089 (0.014) |
|  | LEA |  |  |  |
|  | Diabetic eye disease | 0.003 (0.001) |  |  |
|  | ESRD |  |  | -0.120 (0.012) |
| ARR: Absolute risk reduction; LRR: Ln(Relative Risk).  Mean and SE for ARR and LRR results were converted to per-quarter effects by dividing by 4. Blank cells indicate a lack of evidence.  Effect on BMI was constructed by dividing the effects on weight (in kgs) by 1.65m2. | | | | |

### **Table A8:** Translated quarterly effects of DPP4i and SGLT2i monotherapies, and metformin-DPP4i, and metformin-SGLT2i versus no treatment on various outcomes for the RAPIDS risk engine.

| Biomarkers & Outcomes | Parameter Type | Monotherapy | | Combo Therapy with metformin | |
| --- | --- | --- | --- | --- | --- |
|  |  | DPP4i | SGLT2i | DPP4i | SGLT2i |
| BMI (kg/m2) | ARR | 0.119 (0.014) | -0.173 (0.010) | -0.176 (0.020) | -0.185 (0.009) |
| Haemoglobin A1c (%) | ARR | 0.042 (0.025) | -0.195 (0.021) | -0.026 (0.052) | -0.203 (0.022) |
| High-density lipoprotein (mg/dL) | ARR |  |  |  | 0.013 (0.001) |
| Low-density lipoprotein (mg/dL) | ARR | -0.599 (0.200) | -0.599 (0.200) | -0.599 (0.200) | -0.589 (0.200) |
| Total Cholesterol (mg/dL) | ARR | -0.482 (0.200) | -0.482 (0.200) | -0.482 (0.200) | -0.482 (0.200) |
| Triglyceride (mg/dL) | ARR |  |  |  |  |
| Systolic BP (mm Hg) | ARR |  | -0.615 (0.050) |  | -0.905 (0.076) |
| Diastolic BP (mm Hg) | ARR |  | -0.365 (0.046) |  | -0.365 (0.046) |
| eGFR (mL/min/1.73m2) | ARR | -0.672 (0.300) | -0.535 (0.335) | -0.672 (0.300) | -0.535 (0.335) |
| Death | LRR |  |  |  | -0.035 (0.010) |
| Myocardial Infarction | LRR |  |  |  | -0.026 (0.012) |
| Any unstable angina | LRR |  |  |  |  |
| Stroke | LRR |  |  |  | -0.005 (0.017) |
| Any hypoglycaemia | LRR |  | 0.060 (0.024) | 0.008 (0.055) | 0.060 (0.024) |
| Congestive heart failure | LRR |  | -0.089 (0.014) |  | -0.089 (0.014) |
| Lower extremity amputation | LRR |  |  |  |  |
| Advanced eye disease | LRR |  |  |  |  |
| ESRD | LRR |  | -0.12 (0.012) |  | -0.12 (0.012) |

ARR: Absolute change; LRR: Ln(Relative Risk). Blank cells indicate a lack of evidence which we assume to be a null effect.

Note: Supplementary table 5, ARR effects on Haemoglobin A1c, LDL, Total Cholesterol, and EGFR for DPP4i (mono & combo) & SGLT2i (mono and combo) were converted to be compared to placebo by adding mean effects of Metformin monotherapy versus no treatment estimated quarterly by RAPIDS risk engine (HbA1c: -0.065 (0.020); LDL (-0.599 (0.200); Total Cholesterol: -0.482 (0.200); eGFR: -0.672 (0.300)). Other effects were retained to be the same as in Supplementary table 5, as metformin monotherapy did not have an effect compared to placebo. Mean and SE for ARR and LRR results were converted to per-quarter effects by dividing by 4.

**2.2 Incorporating of other treatment effects from the original (2019) RAPIDS model (Basu et al 2019)**

Treatment effects for the original RAPIDS model were initially estimated using VA data. For each biomarker and long-term event in a given quarter, information in the previous quarter (treatments, demographics, time-updated covariates) were used for a 2-stage residual inclusion approach.^4^ Stage one fitted the treatment indicators against all covariates, and residuals were estimated. Stage 2 fitted a linear or logistic regression (for biomarkers and long-term events, respectively), adjusted for covariates and the residuals estimated in stage 1. We then validated these stage 2 models and used them to predict biomarkers and long-term events in future quarters. For further details of the original RAPIDS model development, please refer to Basu et al. (2019),^5^ and for the antidiabetic treatment effects not of interest to this paper, please refer to the appendix (**Table A3**) of Basu et al 2019. available at

<https://www.dropbox.com/s/s3x5ahd71oua93k/APPENDIXTABLES.xlsx?e=1&dl=0>

### **Table A9:** RAPIDS 90-day quarter treatment effectiveness input for alternative second-line treatments added to metformin versus no treatment.

| **Measure** | **Biomarkers and Long-**  **term outcomes** | **Metformin +** | | |
| --- | --- | --- | --- | --- |
|  |  | **SU** | **DPP4i** | **SGLT2i** |
| Parameter Source | | RAPIDS model | Literature | Literature |
| ARR | BMI (kg/m2) | 0.014 (0.005) | -0.176 (0.020) | -0.185 (0.009) |
|  | HbA1c (%) | -0.151 (0.050) | -0.026 (0.052) | -0.203 (0.022) |
|  | HDL (mg/dL) | -0.210 (0.100) |  | 0.013 (0.001) |
|  | LDL (mg/dL) | -0.872 (0.300) | -0.599 (0.200) | -0.589 (0.200) |
|  | Total Cholesterol (mg/dL) | -0.769 (0.350) | -0.482 (0.200) | -0.482 (0.200) |
|  | Triglycerides (mg/dL) | 1.500 (0.750) |  |  |
|  | SBP (mm Hg) |  |  | -0.905 (0.076) |
|  | DBP (mm Hg) |  |  | -0.365 (0.046) |
|  | eGFR (mL/min/1.73m2) | -0.483 (0.200) | -0.672 (0.300) | -0.535 (0.335) |
| LRR | Death | -0.003 (0.001) |  | -0.035 (0.010) |
|  | MI |  |  | -0.026 (0.012) |
|  | Unstable Angina |  |  |  |
|  | Stroke |  |  | -0.005 (0.017) |
|  | Hypoglycaemia | 0.040 (0.010) | 0.008 (0.055) | 0.060 (0.024) |
|  | HF |  |  | -0.089 (0.014) |
|  | LEA |  |  |  |
|  | Eye disease | 0.003 (0.001) |  |  |
|  | ESRD |  |  | -0.120 (0.012) |
| ARR: Absolute change; LRR: Ln(Relative Risk).  Mean and SE for ARR and LRR results were converted to per-quarter effects by dividing by 4. Blank cells indicate a lack of evidence.  Effect on BMI was constructed by dividing the effects on weight (in kgs) by 1.65m2. | | | | |

# **Stage 3 Model calibration**

## **3.1 Methods**

For each risk factor and complication, we compared the predicted values in each quarter to the corresponding ‘observed’ values from the CPRD cohort. We defined ‘agreement’ as when the observed values were within the 95% predicted intervals. For BMI, total cholesterol and eGFR, this criterion was not met so we re-estimated the parameters in the outcome-specific calibration models of RAPIDS using a random sample of 20,000 (32%) from CPRD with the remainder held-back for out-of-sample validation.

### **Table A10:** Baseline characteristics of the CPRD cohort, overall and stratified by training and validation split

| **Characteristics** | **Total** | **Re-estimation of calibration parameters (training)** | **Validation** |
| --- | --- | --- | --- |
|  | **N=62,640** | **N=20,000** | **N=42,640** |
| **Age (years), median (IQR)** | 60.0 (52.0-69.0) | 60.0 (52.0-69.0) | 60.0 (52.0-69.0) |
| **Female, count (%)** | 24,703 (39.4) | 7,894 (39.5) | 16,809 (39.4) |
| **Ethnicity, count (%)** |  |  |  |
| White | 45,396 (72.5) | 14,520 (72.6) | 30,876 (72.4) |
| Black | 3,125 (5.0) | 957 (4.8) | 2,168 (5.1) |
| Hispanic | 0 (0.0) | 0 (0.0) | 0 (0.0) |
| Other (South Asian, Mixed, Other, missing) | 14,119 (22.5) | 2,852 (14.3) | 9,596 (22.5) |
| **Index of Multiple Deprivation (IMD), count (%)** |  |  |  |
| 1 (least deprived) | 8,866 (14.2) | 2,796 (14.0) | 6,070 (14.2) |
| 2 | 11,098 (17.7) | 3,529 (17.6) | 7,569 (17.8) |
| 3 | 11,330 (18.1) | 3,706 (18.5) | 7,624 (17.9) |
| 4 | 13,729 (21.9) | 4,377 (21.9) | 9,352 (21.9) |
| 5 (most deprived) | 14,502 (23.2) | 4,586 (22.9) | 9,916 (21.9) |
| Missing | 3,115 (5.0) | 1,006 (5.0) | 2,109 (5.0) |
| **Years since diagnosis with T2DM, median (IQR)** | 4.9 (2.5-5.0) | 4.9 (2.5-5.0) | 4.9 (2.5-5.0) |
| **BMI (kg/m^2^), median (IQR)** | 31.3 (28.0-36.0) | 31.3 (28.0-36.0) | 31.4 (28.0-36.0) |
| **Systolic blood pressure (mm Hg), median (IQR)** | 132 (124-140) | 132 (124-140) | 132 (124-140) |
| **Diastolic blood pressure (mm Hg), median (IQR)** | 79.0 (73.0-82.0) | 78.0 (73.0-82.0) | 79.0 (73.0-82.0) |
| **HDL (mmol/L), median (IQR)** | 42.5 (38.7-50.3) | 42.5 (38.7-50.3) | 42.5 (38.7-50.3) |
| **LDL (mmol/L), median (IQR)** | 81.2 (61.9-104.4) | 81.2 (61.9-104.4) | 81.2 (61.9-104.4) |
| **Total cholesterol (mmol/L), median (IQR)** | 158.5 (139.2-185.6) | 158.5 (139.2-185.6) | 158.5 (139.2-185.6) |
| **Triglycerides, non-fasting (mmol/L), median (IQR)** | 159.4 (115.1-221.4) | 159.4 (115.1-221.4) | 159.4 (115.1-221.4) |
| **eGFR (mL/min/1.73m^2^), median (IQR)** | 92 (80-102) | 92 (79-102) | 93.0 (80-102) |
| **HbA1c (%), median (IQR)** | 7.9 (7.3-8.8) | 7.9 (7.3-8.8) | 7.9 (7.3-8.8) |
| **HbA1c (mmol/mol) median (IQR)** | 63 (56-73) | 63 (56-73) | 63 (56-73) |
| **History of any CVD*, count (%)** | 8,642 (13.8) |  |  |
| **History of angina, count (%)** | 2,053 (3.3) | 658 (3.3) | 1,395 (3.3) |
| **History of myocardial infarction, count (%)** | 3,820 (6.1) | 1,220 (6.1) | 2,600 (6.1) |
| **History of stroke, count (%)** | 2,621 (4.2) | 821 (4.1) | 1,800 (4.2) |
| **History of heart failure** | 2,910 (4.7) | 922 (4.6) | 1,988 (4.7) |
| **History of hypoglycaemia, count (%)** | 491 (0.8) | 151 (0.8) | 340 (0.8) |
| IQR: the 25^th^ – 75^th^ percentile  All baseline variables required for RAPIDS 2.0 were available except for marital status and US veteran status. We assumed that 50% of people were married (Supplementary section 1.1) and none were US veterans. IMD was not included in the RAPIDS 2.0 model.  *CVD is a composite of angina, myocardial infarction, stroke, and heart failure | | | |

We specified the calibration model for each outcome as nonlinear mixed-effects regression models between the observed outcome (Y) and the predicted observed outcome ($Y$) and the predicted outcome values without calibration ($\hat{Y}$) at quarter $q$ (equation 1: $E\left[ Y_{q} \right]= \alpha_{0}+ \alpha_{1}^{q} \hat{Y}$).^3^ After regressing the observed versus predicted values, we extracted the intercept and slope parameters ($\alpha_{0},\alpha_{1}$) and bootstrapped them 500 times to update the set of calibration parameters for BMI, total cholesterol, and eGFR for the UK RAPIDS model. We then applied these updated parameters to the remaining 42,640 patients to check for overfitting.

## **3.2 Calibration results**

For most risk factors and all complications, the observed values in the independent hold-out sample fell within the 95% CI of the predictions from the RAPIDS 2.0 model across the full follow-up period (Figure A1). For BMI, total cholesterol, eGFR and DBP (after 7.6 years) the observed values were outside the predicted 95% CI. After calibration adjustment, the observed values for total cholesterol were within the prediction intervals, and for DBP and BMI the remaining discrepancies between the predicted and observed values were small and no further recalibration was undertaken (**Figure A2**).

#### **Figure A1:** Predicted versus observed risk factors (biomarker values) and complications (events) for people who initiate second-line oral glucose-lowering treatment with one of SU, DPP4i, or SGLT2i, added to metformin after applying the US RAPIDS 2.0 calibration model


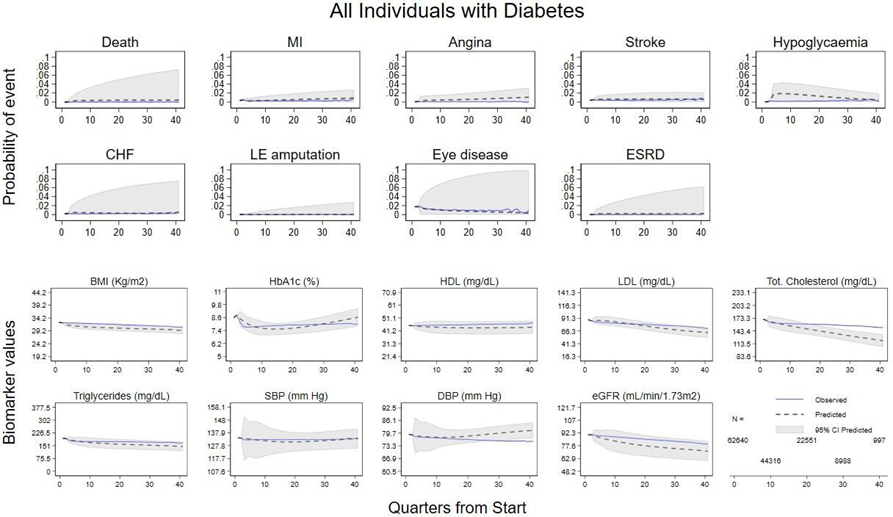


In order to recalibrate the model the data were split into a training and validation set (see Table A10). The RAPIDS model was rerun on the training set without using the RAPIDS 2.0 calibration parameters. The uncalibrated predicted estimates were then compared to the observed values, and the calibration process was implemented. The UK calibrated parameters for BMI, total cholesterol and DBP were used to update the RAPID 2.0 respective parameters and these updated parameters were then evaluated on the 42,540 patients in the validation set. No further calibration updates were required **Figure A2**).

#### **Figure A2:** Predicted versus observed risk factors (biomarker values) and complications (events) for people who initiate second-line oral glucose-lowering treatment with one of SU, DPP4i, or SGLT2i, added to metformin after applying the calibration update to the UK population


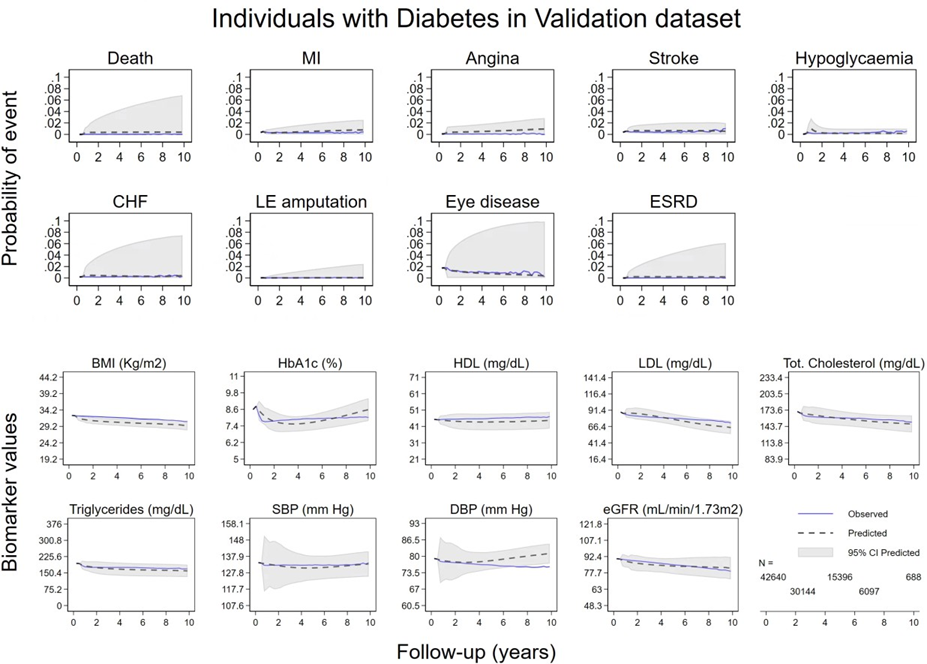


# **Stage 4: Comparison of alternative second-line treatments**

**Evidence to support assumption about duration of second-line treatment**

For the comparison of the second-line treatments on risk factors and complications, we predicted three sets of these outcomes for each person, that is one set following each second-line treatment. As each person only received one of the second-line treatments we were required to make an assumption about the expected duration of second-line treatment and subsequent treatment. The PERMIT study had previously found similar median durations of second-line treatment. Here we assessed whether time to second-line treatment cessation differed across the second-line treatments. We used the same instrumental variable approach (2-stage residual inclusion (2SRI))^4^ as used previously by Bidulka et al (2024) to reduce the risk of bias from unmeasured confounding.^1^

We used a complete-case analysis to handle missing covariate information as this was previously found to produce similar results to using multiple imputation in the PERMIT cohort.^1^ We used clinician preference as an instrument for treatment prescription,^15,16^ specifically using the preference at the clinical commissioning groups (CCGs) level, who inform prescribing decisions for participating general practices (further details for instrument choice and assumptions available in Bidulka et al, 2024).^1^

The two first stage logistic regression models (the outcome for the first model is an indicator of having been prescribed DPP4i and the second model is an outcome for being prescribed SGLT2i) estimated the probabilities that each person was prescribed either treatment given baseline covariates and the instrument. The second stage model used a Cox proportional hazards model which account for individual frailty^17^ for time to first treatment switching, which incorporated generalised residuals from the two first stage models, alongside baseline covariates. The baseline covariates for the stage 1 and 2 models are: baseline age, sex, ethnicity, index of multiple deprivation, days since 2^nd^ line treatment initiation, practice size in 2014, renin-angiotensin system inhibitors (RASI) or statins prescribed in the 60 days prior to the index date, baseline HbA1c, baseline systolic blood pressure, baseline diastolic blood pressure, baseline eGFR, baseline BMI, smoking status, alcohol status, year of 2^nd^ line treatment initiation, hospitalization in the year prior to the index date, and a history of myocardial infarction, unstable angina, stroke, ischaemic heart disease, hypoglycaemia, heart failure, cancer, proteinuria, advanced eye disease, lower extremity amputation, or chronic kidney disease.

The results below (Table A11) indicate that there was no evidence of differences in time to second-line treatment cessation. Hence, in predicting the counterfactual outcomes we assumed that for an individual the time to stopping second-line treatment was for the for the three alternative treatments, and also that the subsequent treatments were the same as in the observed data. This approach did allow for the duration of second-line treatment and choice of subsequent treatments to differ across individuals, according to the treatment duration and subsequent pathway observed for that individual.

### **Table A11:** Assessment of the time to second-line glucose-lowering treatment cessation across the alternative second-line treatments (N=54,033)

| Comparison | Hazard Ratio | 95% CI | P-value for Ho:  𝜷𝑺𝑮𝑳𝑻𝟐𝒊 − 𝜷𝑫𝑷𝑷𝟒𝒊 =0 | P-value for Ho:  𝜷𝑫𝑷𝑷𝟒𝒊 – 𝜷SU =0 |
| --- | --- | --- | --- | --- |
| SGLT2i vs SU | 0.89 | (0.79, 1.01) | 0.060 | 0.138 |
| DPP4i vs SU | 1.02 | (0.93, 1.12) |  |  |

Cox proportional hazards was adjusted for:

Baseline age, sex, ethnicity, index of multiple deprivation, Days since 2nd line treatment assignment, Practice size in 2014, Renin-Angiotensin System Inhibitors (RASi), Statin, Baseline HbA1c, Baseline systolic blood pressure, Baseline diastolic blood pressure, Baseline eGFR, Baseline BMI, smoking status, alcohol status (non-drinker, ex-drinker, current drinker), year of first 2 line initiation, IHD, hospital attendance in last year, and a history of myocardial infarction, unstable angina, stroke, hypoglycaemia, heart Failure, cancer history, history of proteinuria, advanced eye disease, lower extremity amputation or chronic kidney disease

### **Table A12:** Baseline characteristics of patients with type 2 diabetes mellitus 2011-2020 from the CPRD eligible cohort, stratified by 2^nd^ line treatment initiated, added to metformin

| **Characteristics** | **Total** | **Metformin &**  **SU** | **Metformin &**  **DPP4i** | **Metformin &**  **SGLT2i** |
| --- | --- | --- | --- | --- |
|  | **N=62,640** | **N=30,704** | **N=25,539** | **N=6,397** |
| *Demographics and biomarkers* | | | | |
| Age (years), median (IQR) | 60.0 (52.0-69.0) | 61.0 (52.0-69.0) | 61.0 (53.0-69.0) | 56.0 (49.0-63.0) |
| Female | 24,703 (39.4) | 12,090 (39.4) | 10,042 (39.3) | 2,571 (40.2) |
| **Ethnicity, count (%)** |  |  |  |  |
| White | 45,396 (72.5) | 21,645 (70.5) | 18,933 (74.1) | 4,818 (75.3) |
| Black | 3,125 (5.0) | 1,819 (5.9) | 1,069 (4.2) | 237 (3.7) |
| Other (South Asian,  Mixed, Other, missing) | 14,119 (22.5) | 7,240 (23.6) | 5,537 (21.7) | 1,342 (21.0) |
| **Index of Multiple**  **Deprivation (IMD), count (%)** |  |  |  |  |
| 1 (least deprived) | 8,866 (14.2) | 4,320 (14.1) | 3,662 (14.3) | 884 (13.8) |
| 2 | 11,098 (17.7) | 5,339 (17.4) | 4,664 (18.3) | 1,095 (17.1) |
| 3 | 11,330 (18.1) | 5,527 (18.0) | 4,643 (18.2) | 1,160 (18.1) |
| 4 | 13,729 (21.9) | 6,732 (21.9) | 5,554 (21.8) | 1,443 (22.5) |
| 5 (most deprived) | 14,502 (23.2) | 6,960 (22.7) | 6,032 (23.6) | 1,510 (23.6) |
| Missing | 3,115 (5.0) | 1,826 (6.0) | 984 (3.9) | 305 (4.8) |
| Years since T2DM  diagnosis, median (IQR) | 4.9 (2.5-5.0) | 4.8 (2.3-5.0) | 5.0 (2.7-5.0) | 4.5 (2.3-5.0) |
| BMI (kg/m2), median (IQR) | 31.3 (28.0-36.0) | 31.0 (27.0-35.0) | 31.8 (28.0-36.0) | 34.0 (30.0-39.0) |
| Systolic blood pressure  (mm Hg), median (IQR) | 132.0 (124.0-  140.0) | 132.0 (124.0-  140.0) | 132.0 (124.0-  140.0) | 131.0 (124.0-  139.0) |
| Diastolic blood pressure  (mm Hg), median (IQR) | 79.0 (73.0-82.0) | 78.0 (72.0-82.0) | 78.0 (73.0-82.0) | 80.0 (75.0-83.0) |
| HDL (mmol/L), median  (IQR) | 42.5 (38.7-50.3) | 42.5 (38.7-50.3) | 42.5 (38.7-50.3) | 42.5 (38.7-50.3) |
| LDL (mmol/L), median  (IQR) | 81.2 (61.9-104.4) | 81.2 (61.9-104.4) | 77.3 (61.9-100.5) | 81.2 (61.9-104.4) |
| Total cholesterol  (mmol/L), median (IQR) | 158.5 (139.2-  185.6) | 162.4 (139.2-  185.6) | 158.5 (135.3-  181.7) | 162.4 (139.2-  189.5) |
| Triglycerides, random  (mmol/L), median (IQR) | 159.4 (115.1-  221.4) | 159.4 (115.1-  221.4) | 159.4 (115.1-  221.4) | 177.1 (124.0-  230.3) |
| eGFR (mL/min/1.73m2),  median (IQR) | 92.0 (80.0-102.0) | 92.0 (79.0-102.0) | 91.0 (78.0-101.0) | 97.0 (87.0-105.0) |
| HbA1c (%), median (IQR) | 7.9 (7.3-8.8) | 7.9 (7.3-9.0) | 7.8 (7.3-8.6) | 7.9 (7.3-8.7) |
| *Long-term events* | | | | |
| History of CVD* | 8,642 (13.8) | 4,392 (14.3) | 3,580 (14.0) | 670 (10.5) |
| History of angina | 2,053 (3.3) | 1,048 (3.4) | 864 (3.4) | 141 (2.2) |
| History of myocardial  Infarction | 3,820 (6.1) | 1,922 (6.3) | 1,588 (6.2) | 310 (4.8) |
| History of stroke | 2,621 (4.2) | 1,355 (4.4) | 1,062 (4.2) | 204 (3.2) |
| History of heart failure | 2,910 (4.7) | 1,466 (4.8) | 1,236 (4.8) | 2,910 (4.7) |
| History of hypoglycaemia | 491 (0.8) | 240 (0.8) | 201 (0.8) | 50 (0.8) |

CVD: cardiovascular disease; DPP4i: dipeptidyl peptidase-4 inhibitor; eGFR: estimated glomerular filtration rate; IQR: Interquartile range; SGLT2i: sodium-glucose co-transporter 2 inhibitor; SU: sulfonylurea; T2DM: type 2 diabetes mellitus

*CVD is a composite of angina, myocardial infarction, stroke, and heart failure

### **Table A13:** Observed glucose-lowering treatment courses (proportion of people prescribed) over each year of follow-up included in the RAPIDS model categorised according to the Sankey plot (main text Figure 1)

|  |  | **Year** | | | | | | | |
| --- | --- | --- | --- | --- | --- | --- | --- | --- | --- |
|  | **ANTIDIABETIC DRUG(S)** | **0** | **1** | **2** | **3** | **4** | **5** | **6** | **7** |
| **No. of drugs** | **N contributing data to the cohort** | **62,640** | **57,951** | **46,726** | **36,814** | **28,170** | **20,917** | **14,988** | **10,035** |
| 2 | 1. Metformin-SU | 49.3 | 33.6 | 30.2 | 27.3 | 24.6 | 22.2 | 19.3 | 15.6 |
| 2 | 2. Metformin-DPP4i | 40.8 | 28.1 | 23.3 | 19.6 | 16.7 | 14.4 | 13.0 | 12.1 |
| 2 | 3. Metformin-SGLT2i | 9.9 | 6.5 | 5.4 | 4.6 | 4.3 | 3.8 | 3.3 | 3.7 |
| 2 | 4. Metformin-GLP1-RA | 0.0 | 0.9 | 1.3 | 1.5 | 1.6 | 1.6 | 1.7 | 1.8 |
| 1 | 5. Metformin monotherapy | 0.0 | 12.4 | 12.6 | 12.5 | 12.3 | 12.1 | 12.3 | 11.8 |
| 1 | 6. Other monotherapy | 0.0 | 4.4 | 4.4 | 4.6 | 4.9 | 5.0 | 5.3 | 5.8 |
| 2 | 7. Other duotherapy | 0.0 | 1.6 | 2.8 | 3.7 | 4.6 | 5.4 | 6.4 | 6.9 |
| 3 | 8. Any triple therapy | 0.0 | 7.4 | 13.9 | 19.3 | 23.5 | 27.1 | 29.8 | 30.8 |
| ≥4 | 9. Any 4+ therapy | 0.0 | 0.2 | 0.6 | 1.2 | 1.9 | 2.5 | 3.2 | 4.3 |
| 0 | 10. No treatment | 0.0 | 5.0 | 5.5 | 5.8 | 5.8 | 5.9 | 5.8 | 7.3 |

DPP4i: dipeptidyl peptidase-4 inhibitor; GLP1-RA: glucagon-like peptide-1 receptor agonist; SGLT2i: sodium-glucose co-transporter 2 inhibitor; SU: sulfonylurea

### **Table A14:** Observed glucose-lowering treatment courses (proportion of people prescribed) over each year of follow-up in the RAPIDS model

|  |  | **YEAR** |  |  |  |  |  |  |  |
| --- | --- | --- | --- | --- | --- | --- | --- | --- | --- |
|  | **ANTIDIABETIC DRUG(S)** | **0** | **1** | **2** | **3** | **4** | **5** | **6** | **7** |
| **No. of drugs** | **N contributing data to the cohort** | **62,640** | **57,951** | **46,726** | **36,814** | **28,170** | **20,917** | **14,988** | **10,035** |
| 0 | No treatment | 0.0 | 5.0 | 5.5 | 5.8 | 5.8 | 5.9 | 5.8 | 7.3 |
| 1 | Acarbose | 0.0 | 0.0 | 0.0 | 0.0 | 0.0 | 0.0 | 0.0 | 0.0 |
| 1 | DPP4i | 0.0 | 1.5 | 1.3 | 1.3 | 1.3 | 1.4 | 1.4 | 1.6 |
| 1 | Glinide | 0.0 | 0.0 | 0.0 | 0.0 | 0.0 | 0.0 | 0.0 | 0.0 |
| 1 | GLP1-RA | 0.0 | 0.1 | 0.2 | 0.2 | 0.2 | 0.2 | 0.2 | 0.3 |
| 1 | Insulin | 0.0 | 0.3 | 0.5 | 0.7 | 1.0 | 1.2 | 1.6 | 1.8 |
| 1 | Metformin | 0.0 | 12.4 | 12.6 | 12.5 | 12.3 | 12.1 | 12.3 | 11.8 |
| 1 | SGLT2i | 0.0 | 0.4 | 0.4 | 0.3 | 0.3 | 0.4 | 0.3 | 0.4 |
| 1 | SU | 0.0 | 2.1 | 2.0 | 2.0 | 2.0 | 1.8 | 1.8 | 1.6 |
| 1 | TZD | 0.0 | 0.0 | 0.0 | 0.0 | 0.1 | 0.0 | 0.0 | 0.1 |
| 2 | DPP4i, acarbose | 0.0 | 0.0 | 0.0 | 0.0 | 0.0 | 0.0 | 0.0 | 0.0 |
| 2 | DPP4i, GLP1-RA | 0.0 | 0.0 | 0.0 | 0.0 | 0.0 | 0.0 | 0.0 | 0.0 |
| 2 | DPP4i, Glinide | 0.0 | 0.0 | 0.0 | 0.0 | 0.0 | 0.0 | 0.0 | 0.0 |
| 2 | DPP4i, insulin | 0.0 | 0.0 | 0.1 | 0.1 | 0.2 | 0.3 | 0.4 | 0.4 |
| 2 | DPP4i, TZD | 0.0 | 0.0 | 0.0 | 0.0 | 0.0 | 0.0 | 0.0 | 0.0 |
| 2 | DPP4i, SGLT2i | 0.0 | 0.1 | 0.2 | 0.2 | 0.2 | 0.2 | 0.2 | 0.2 |
| 2 | GLP1-RA, Glinide | 0.0 | 0.0 | 0.0 | 0.0 | 0.0 | 0.0 | 0.0 | 0.0 |
| 2 | GLP1-RA, insulin | 0.0 | 0.0 | 0.0 | 0.0 | 0.1 | 0.1 | 0.2 | 0.2 |
| 2 | GLP1-RA, TZD | 0.0 | 0.0 | 0.0 | 0.0 | 0.0 | 0.0 | 0.0 | 0.0 |
| 2 | Insulin, arcabose | 0.0 | 0.0 | 0.0 | 0.0 | 0.0 | 0.0 | 0.0 | 0.0 |
| 2 | Metformin, arcabose | 0.0 | 0.0 | 0.0 | 0.0 | 0.0 | 0.0 | 0.0 | 0.0 |
| 2 | Metformin, DPP4i | 40.8 | 28.1 | 23.3 | 19.6 | 16.7 | 14.4 | 13.0 | 12.1 |
| 2 | Metformin, Glinide | 0.0 | 0.0 | 0.0 | 0.0 | 0.0 | 0.0 | 0.0 | 0.0 |
| 2 | Metformin, GLP1-RA | 0.0 | 0.9 | 1.3 | 1.5 | 1.6 | 1.6 | 1.7 | 1.8 |
| 2 | Metformin, insulin | 0.0 | 0.5 | 1.0 | 1.4 | 1.9 | 2.3 | 2.9 | 3.2 |
| 2 | Metformin, SGLT2i | 9.9 | 6.5 | 5.4 | 4.6 | 4.3 | 3.8 | 3.3 | 3.7 |
| 2 | Metformin, SU | 49.3 | 33.6 | 30.2 | 27.3 | 24.6 | 22.2 | 19.3 | 15.6 |
| 2 | Metformin, TZD | 0.0 | 0.3 | 0.3 | 0.4 | 0.4 | 0.4 | 0.4 | 0.4 |
| 2 | SGLT2i, acarbose | 0.0 | 0.0 | 0.0 | 0.0 | 0.0 | 0.0 | 0.0 | 0.0 |
| 2 | SGLT2i, Glinide | 0.0 | 0.0 | 0.0 | 0.0 | 0.0 | 0.0 | 0.0 | 0.0 |
| 2 | SGLT2i, GLP1-RA | 0.0 | 0.0 | 0.0 | 0.0 | 0.0 | 0.0 | 0.1 | 0.1 |
| 2 | SGLT2i, insulin | 0.0 | 0.0 | 0.0 | 0.0 | 0.0 | 0.1 | 0.1 | 0.1 |
| 2 | SGLT2i, TZD | 0.0 | 0.0 | 0.0 | 0.0 | 0.0 | 0.0 | 0.0 | 0.0 |
| 2 | SU, acarbose | 0.0 | 0.0 | 0.0 | 0.0 | 0.0 | 0.0 | 0.0 | 0.0 |
| 2 | SU, DPP4i | 0.0 | 0.4 | 0.8 | 1.0 | 1.1 | 1.3 | 1.3 | 1.4 |
| 2 | SU, Glinide | 0.0 | 0.0 | 0.0 | 0.0 | 0.0 | 0.0 | 0.0 | 0.0 |
| 2 | SU, GLP1-RA | 0.0 | 0.0 | 0.1 | 0.1 | 0.1 | 0.1 | 0.2 | 0.1 |
| 2 | SU, insulin | 0.0 | 0.1 | 0.1 | 0.1 | 0.1 | 0.2 | 0.2 | 0.3 |
| 2 | SU, SGLT2i | 0.0 | 0.0 | 0.1 | 0.1 | 0.2 | 0.2 | 0.3 | 0.4 |
| 2 | SU, TZD | 0.0 | 0.0 | 0.0 | 0.0 | 0.1 | 0.1 | 0.1 | 0.0 |
| 2 | TZD, glinide | 0.0 | 0.0 | 0.0 | 0.0 | 0.0 | 0.0 | 0.0 | 0.0 |
| 2 | TZD, insulin | 0.0 | 0.0 | 0.0 | 0.0 | 0.0 | 0.0 | 0.0 | 0.0 |
| 3 | DPP4i, SGLT2i, GLP1-RA | 0.0 | 0.0 | 0.0 | 0.0 | 0.0 | 0.0 | 0.0 | 0.0 |
| 3 | DPP4i, SGLT2i, Insulin | 0.0 | 0.0 | 0.0 | 0.0 | 0.0 | 0.0 | 0.0 | 0.0 |
| 3 | Metformin, DPP4i, GLP1-RA | 0.0 | 0.1 | 0.2 | 0.2 | 0.2 | 0.2 | 0.2 | 0.2 |
| 3 | Metformin, DPP4i, SGLT2i | 0.0 | 1.4 | 2.4 | 3.1 | 3.5 | 3.6 | 3.8 | 4.2 |
| 3 | Metformin, SGLT2i, Insulin | 0.0 | 0.0 | 0.1 | 0.2 | 0.3 | 0.4 | 0.6 | 0.8 |
| 3 | Metformin, SGLT2i, GLP1-RA | 0.0 | 0.1 | 0.2 | 0.3 | 0.6 | 0.8 | 0.9 | 1.2 |
| 3 | Metformin, SU, DPP4i | 0.0 | 4.1 | 7.5 | 10.0 | 11.7 | 12.7 | 13.4 | 12.6 |
| 3 | Metformin, SU, GLP1-RA | 0.0 | 0.3 | 0.7 | 1.1 | 1.3 | 1.6 | 1.6 | 1.7 |
| 3 | Metformin, SU, SGLT2i | 0.0 | 0.5 | 1.2 | 1.9 | 2.8 | 3.8 | 4.6 | 5.2 |
| 3 | Metformin, DPP4i, Insulin | 0.0 | 0.1 | 0.2 | 0.4 | 0.5 | 0.7 | 0.9 | 1.1 |
| 3 | Metformin, DPP4i, TZD | 0.0 | 0.2 | 0.4 | 0.5 | 0.5 | 0.5 | 0.6 | 0.6 |
| 3 | Metformin, GLP1-RA, TZD | 0.0 | 0.0 | 0.0 | 0.0 | 0.0 | 0.0 | 0.0 | 0.0 |
| 3 | Metformin, SGLT2i, Insulin | 0.0 | 0.0 | 0.1 | 0.2 | 0.3 | 0.4 | 0.7 | 0.9 |
| 3 | Metformin, SGLT2i, TZD | 0.0 | 0.0 | 0.0 | 0.1 | 0.1 | 0.2 | 0.2 | 0.2 |
| 3 | Metformin, SU, Insulin | 0.0 | 0.2 | 0.3 | 0.5 | 0.6 | 0.8 | 0.8 | 0.8 |
| 3 | Metformin, SU, TZD | 0.0 | 0.2 | 0.3 | 0.4 | 0.5 | 0.6 | 0.6 | 0.5 |
| 3 | SU, DPP4i, Insulin | 0.0 | 0.0 | 0.0 | 0.1 | 0.1 | 0.1 | 0.3 | 0.2 |
| 3 | SU, DPP4i, SGLT2i | 0.0 | 0.0 | 0.0 | 0.1 | 0.1 | 0.2 | 0.2 | 0.2 |
| 3 | SU, SGLT2i, GLP1-RA | 0.0 | 0.0 | 0.0 | 0.0 | 0.0 | 0.0 | 0.1 | 0.0 |
| 3 | Any other combination | 0.0 | 0.1 | 0.1 | 0.2 | 0.2 | 0.3 | 0.3 | 0.3 |
| 4 | Any combination | 0.0 | 0.2 | 0.5 | 1.1 | 1.8 | 2.4 | 3.2 | 4.2 |
| 5 | Any combination | 0.0 | 0.0 | 0.0 | 0.0 | 0.1 | 0.1 | 0.1 | 0.1 |
| ≥6 | Any combination | 0.0 | 0.0 | 0.0 | 0.0 | 0.0 | 0.0 | 0.0 | 0.0 |

DPP4i: dipeptidyl peptidase-4 inhibitor; GLP1-RA: glucagon-like peptide-1 receptor agonist; SGLT2i: sodium-glucose co-transporter 2 inhibitor; SU: sulfonylurea; TZD: thiazolidinedione

#### **Figure A3:** Figure showing the predicted mean absolute values for HbA1c (%), eGFR (mL/min/1.73m2), SBP (mm Hg), and BMI (kg/m2) from the RAPIDS-UK model. The solid black line shows the predicted mean values and the dotted lines represent 95% confidence intervals. A range in HbA1c of 7.5-8.5% is equivalent to 58-69mmol/mol.

**
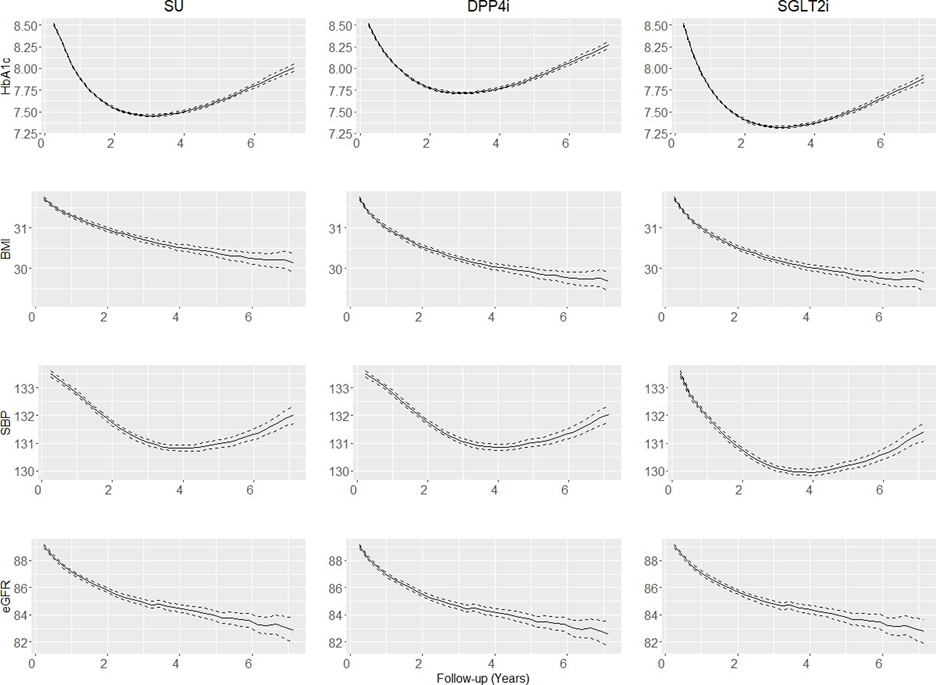
**

#### **Figure A4:** Figure showing the predicted mean absolute values for HDL (mg/dL), LDL (mg/dL), total cholesterol (mg/dL), triglycerides (mg/dL), and DBP (mm Hg) in the RAPIDS model. The solid black line shows the predicted mean values and the dotted lines represent 95% confidence intervals.


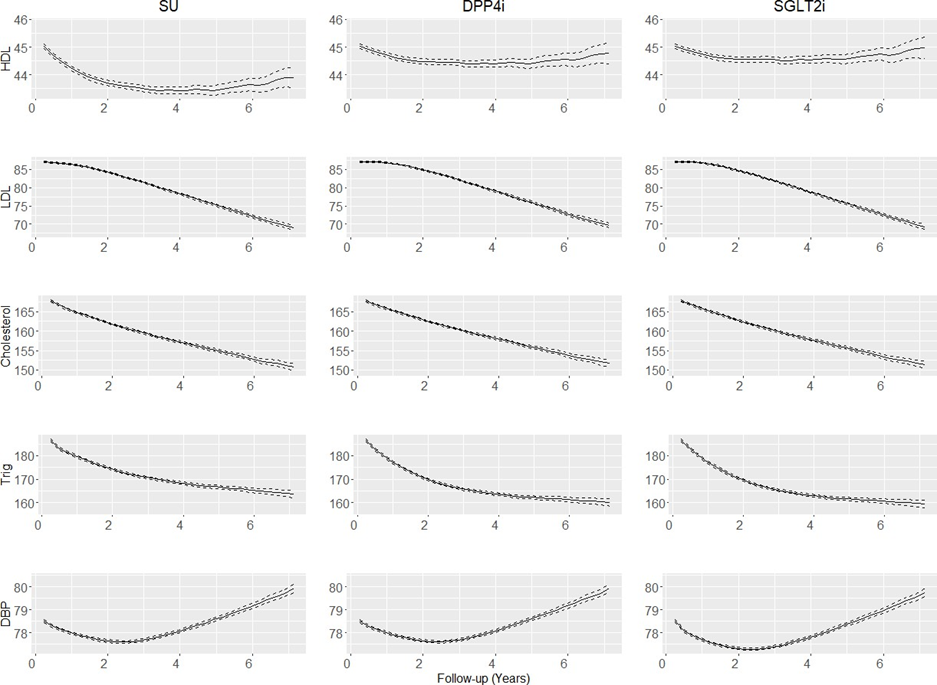


### **Table A15:** Five-year estimates of the predicted mean values of the risk factors (biomarkers) under each counterfactual pathway

| **Biomarkers** | **Counterfactual** | **Estimate** | **95% Confidence Interval** | |
| --- | --- | --- | --- | --- |
| BMI (kg/m2) | SU | 30.40 | 30.30 | 30.50 |
|  | DPP4i | 29.92 | 29.83 | 30.02 |
|  | SGLT2i | 29.91 | 29.81 | 30.01 |
| HbA1c (%) | SU | 7.62 | 7.60 | 7.64 |
|  | DPP4i | 7.89 | 7.87 | 7.90 |
|  | SGLT2i | 7.49 | 7.47 | 7.51 |
| HDL (mg/dL) | SU | 43.43 | 43.27 | 43.59 |
|  | DPP4i | 44.40 | 44.24 | 44.56 |
|  | SGLT2i | 44.56 | 44.40 | 44.72 |
| LDL (mg/dL) | SU | 75.46 | 75.15 | 75.77 |
|  | DPP4i | 76.18 | 75.87 | 76.50 |
|  | SGLT2i | 75.87 | 75.56 | 76.18 |
| Total cholesterol (mg/dL) | SU | 155.01 | 154.61 | 155.41 |
|  | DPP4i | 155.95 | 155.55 | 156.35 |
|  | SGLT2i | 155.61 | 155.21 | 156.01 |
| Triglycerides (mg/dL) | SU | 166.77 | 166.07 | 167.47 |
|  | DPP4i | 162.32 | 161.62 | 163.02 |
|  | SGLT2i | 161.64 | 160.94 | 162.34 |
| SBP (mm Hg) | SU | 130.97 | 130.83 | 131.11 |
|  | DPP4i | 131.01 | 130.87 | 131.15 |
|  | SGLT2i | 130.19 | 130.05 | 130.32 |
| DBP (mm Hg) | SU | 78.57 | 78.50 | 78.65 |
|  | DPP4i | 78.58 | 78.50 | 78.66 |
|  | SGLT2i | 78.35 | 78.27 | 78.42 |
| eGFR  (mL/min/1.73m2) | SU | 83.97 | 83.60 | 84.35 |
|  | DPP4i | 83.69 | 83.31 | 84.07 |
|  | SGLT2i | 83.88 | 83.50 | 84.26 |

#### **Figure A5:** Mean difference in the predicted mean levels of for HbA1c (%), eGFR (mL/min/1.73m2), SBP (mm Hg), and BMI (kg/m2) (solid black lines) across counterfactual second-line oral antidiabetic treatment scenarios: DPP4i vs. SU; SGLT2i vs. SU; SGLT2i vs. DPP4i. The 95% confidence intervals for the difference in predicted probabilities are also presented (dashed black lines). A change in HbA1c of ±0.2% is equivalent to ±2.2mmol/mol.


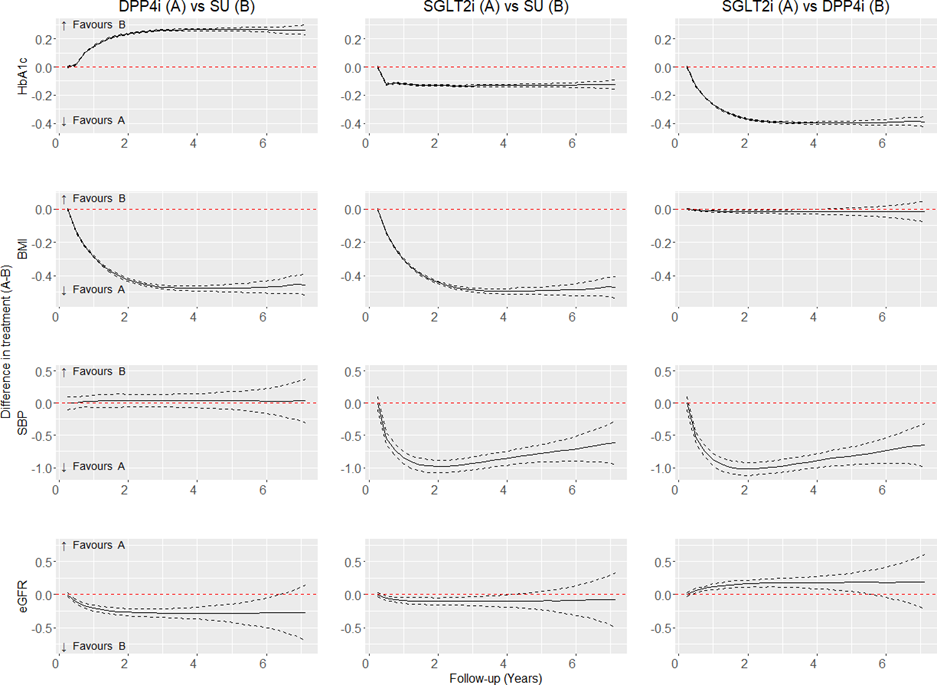


#### **Figure A6:** Figure showing the mean difference in the predicted mean values for HDL (mg/dL), LDL (mg/dL), total cholesterol (mg/dL), triglycerides (mg/dL), and DBP (mm Hg), compared across counterfactual second-line oral antidiabetic treatment scenarios. The solid black lines show the mean difference and the dotted lines the 95% confidence interval.


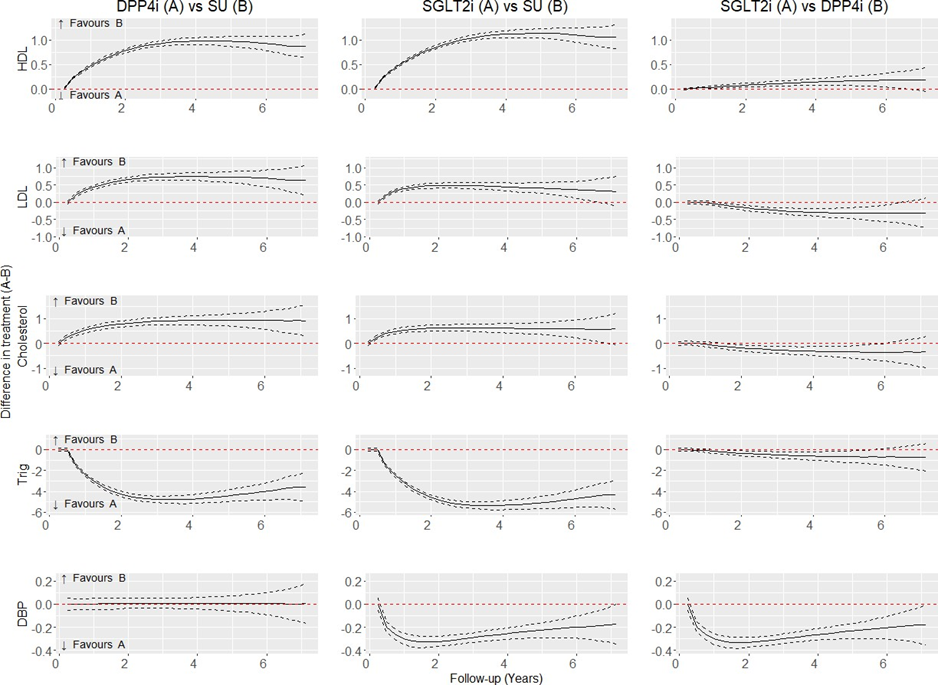


### **Table A16:** Five-year estimates of the mean difference in predicted values of the risk factors (biomarkers) across counterfactual second-line oral antidiabetic treatment scenarios

| **Biomarkers** | **Comparison** | **Estimate** | **95% Confidence Interval** | |
| --- | --- | --- | --- | --- |
| BMI (kg/m2) | DPP4i vs SU | -0.47 | -0.50 | -0.45 |
|  | SGLT2i vs SU | -0.49 | -0.51 | -0.47 |
|  | SGLT2i vs DPP4i | -0.02 | -0.04 | 0.00 |
| HbA1c (%) | DPP4i vs SU | 0.27 | 0.26 | 0.28 |
|  | SGLT2i vs SU | -0.13 | -0.14 | -0.12 |
|  | SGLT2i vs DPP4i | -0.40 | -0.41 | -0.38 |
| HDL (mg/dL) | DPP4i vs SU | 0.97 | 0.88 | 1.07 |
|  | SGLT2i vs SU | 1.13 | 1.03 | 1.23 |
|  | SGLT2i vs DPP4i | 0.16 | 0.07 | 0.25 |
| LDL (mg/dL) | DPP4i vs SU | 0.73 | 0.57 | 0.88 |
|  | SGLT2i vs SU | 0.41 | 0.25 | 0.57 |
|  | SGLT2i vs DPP4i | -0.32 | -0.47 | -0.16 |
| Total cholesterol (mg/dL) | DPP4i vs SU | 0.94 | 0.70 | 1.17 |
|  | SGLT2i vs SU | 0.60 | 0.36 | 0.83 |
|  | SGLT2i vs DPP4i | -0.34 | -0.58 | -0.11 |
| Triglycerides (mg/dL) | DPP4i vs SU | -4.44 | -5.00 | -3.89 |
|  | SGLT2i vs SU | -5.12 | -5.68 | -4.57 |
|  | SGLT2i vs DPP4i | -0.68 | -1.22 | -0.14 |
| SBP (mm Hg) | DPP4i vs SU | 0.04 | -0.09 | 0.18 |
|  | SGLT2i vs SU | -0.78 | -0.92 | -0.65 |
|  | SGLT2i vs DPP4i | -0.82 | -0.96 | -0.69 |
| DBP (mm Hg) | DPP4i vs SU | 0.01 | -0.06 | 0.07 |
|  | SGLT2i vs SU | -0.23 | -0.29 | -0.16 |
|  | SGLT2i vs DPP4i | -0.24 | -0.30 | -0.17 |
| eGFR  (mL/min/1.73m2) | DPP4i vs SU | -0.28 | -0.42 | -0.15 |
|  | SGLT2i vs SU | -0.09 | -0.23 | 0.04 |
|  | SGLT2i vs DPP4i | 0.19 | 0.05 | 0.32 |

#### **Figure A7:** Figure showing the predicted mean absolute probabilities for ESRD, MI, LE amputation and HF from the RAPIDS model. The solid black line shows the predicted mean values and the dotted lines represent 95% confidence intervals.

**
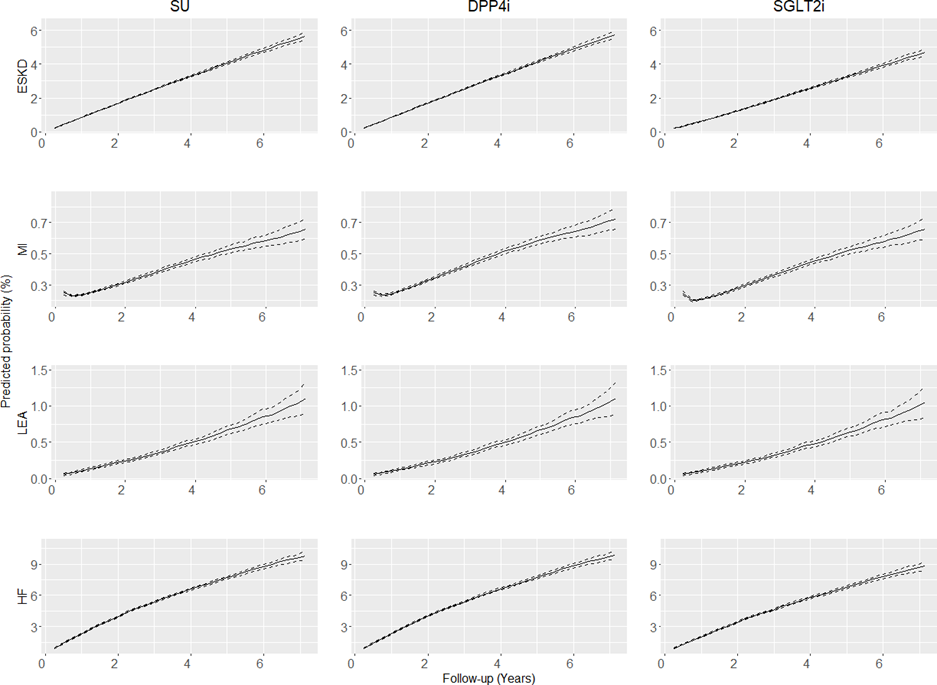
**

#### **Figure A8:** Figure showing the predicted mean probabilities for all-cause death, angina, stroke, diabetic eye disease, and hypoglycaemia in the RAPIDS model. The solid black line shows the predicted mean values and the dotted lines represent 95% confidence intervals.

**
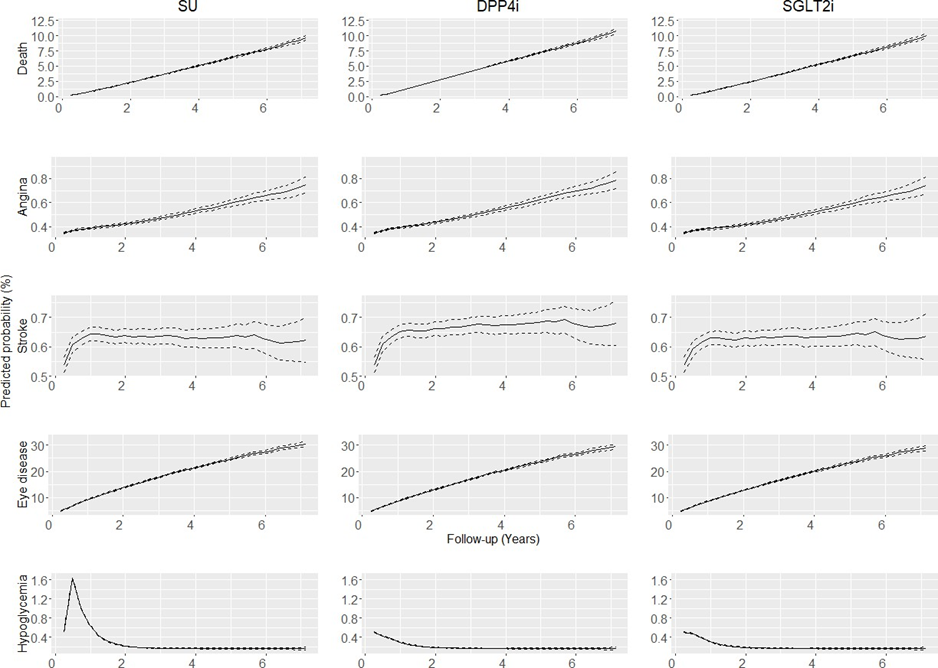
**

### **Table A17:** Five-year estimates of the mean predicted probabilities of complications under each counterfactual pathway

| **Events** | **Counterfactual** | **Estimate** | **95% Confidence**  **Interval** | |
| --- | --- | --- | --- | --- |
| All-cause death | SU | 6.34 | 6.22 | 6.46 |
|  | DPP4i | 7.23 | 7.12 | 7.35 |
|  | SGLT2i | 6.63 | 6.51 | 6.75 |
| MI | SU | 0.53 | 0.50 | 0.55 |
|  | DPP4i | 0.58 | 0.56 | 0.61 |
|  | SGLT2i | 0.52 | 0.50 | 0.54 |
| Angina | SU | 0.59 | 0.56 | 0.62 |
|  | DPP4i | 0.62 | 0.60 | 0.65 |
|  | SGLT2i | 0.58 | 0.56 | 0.61 |
| Stroke | SU | 0.63 | 0.60 | 0.67 |
|  | DPP4i | 0.68 | 0.65 | 0.72 |
|  | SGLT2i | 0.64 | 0.60 | 0.68 |
| HF | SU | 7.62 | 7.48 | 7.75 |
|  | DPP4i | 7.66 | 7.52 | 7.80 |
|  | SGLT2i | 6.71 | 6.58 | 6.85 |
| LE amputation | SU | 0.67 | 0.61 | 0.73 |
|  | DPP4i | 0.66 | 0.60 | 0.72 |
|  | SGLT2i | 0.63 | 0.57 | 0.69 |
| Diabetic Eye disease | SU | 24.41 | 23.98 | 24.83 |
|  | DPP4i | 23.44 | 23.01 | 23.86 |
|  | SGLT2i | 23.00 | 22.57 | 23.42 |
| Hypoglycemia | SU | 0.17 | 0.16 | 0.18 |
|  | DPP4i | 0.17 | 0.16 | 0.18 |
|  | SGLT2i | 0.17 | 0.16 | 0.18 |
| ESRD | SU | 4.01 | 3.95 | 4.08 |
|  | DPP4i | 4.07 | 4.01 | 4.14 |
|  | SGLT2i | 3.20 | 3.13 | 3.27 |

### **Table A18:** Five-year estimates of the mean difference in predicted mean values of the complications across counterfactual second-line oral antidiabetic treatment scenarios.

| **Events** | **Comparison** | **Estimate** | **95% CI** | |
| --- | --- | --- | --- | --- |
| All-cause death | DPP4i vs SU | 0.90 | 0.80 | 0.99 |
|  | SGLT2i vs SU | 0.29 | 0.20 | 0.38 |
|  | SGLT2i vs DPP4i | -0.61 | -0.70 | -0.51 |
| MI | DPP4i vs SU | 0.06 | 0.03 | 0.09 |
|  | SGLT2i vs SU | -0.01 | -0.03 | 0.02 |
|  | SGLT2i vs DPP4i | -0.06 | -0.09 | -0.04 |
| Angina | DPP4i vs SU | 0.03 | 0.00 | 0.06 |
|  | SGLT2i vs SU | -0.01 | -0.03 | 0.02 |
|  | SGLT2i vs DPP4i | -0.04 | -0.07 | -0.01 |
| Stroke | DPP4i vs SU | 0.05 | 0.02 | 0.08 |
|  | SGLT2i vs SU | 0.01 | -0.02 | 0.04 |
|  | SGLT2i vs DPP4i | -0.04 | -0.07 | -0.01 |
| HF | DPP4i vs SU | 0.04 | -0.06 | 0.15 |
|  | SGLT2i vs SU | -0.90 | -1.01 | -0.80 |
|  | SGLT2i vs DPP4i | -0.95 | -1.06 | -0.84 |
| LE amputation | DPP4i vs SU | -0.01 | -0.04 | 0.02 |
|  | SGLT2i vs SU | -0.03 | -0.06 | -0.01 |
|  | SGLT2i vs DPP4i | -0.03 | -0.05 | 0.00 |
| Diabetic Eye disease | DPP4i vs SU | -0.97 | -1.13 | -0.82 |
|  | SGLT2i vs SU | -1.41 | -1.57 | -1.26 |
|  | SGLT2i vs DPP4i | -0.44 | -0.59 | -0.29 |
| Hypoglycemia | DPP4i vs SU | 0.00 | -0.02 | 0.01 |
|  | SGLT2i vs SU | 0.00 | -0.02 | 0.02 |
|  | SGLT2i vs DPP4i | 0.00 | -0.01 | 0.02 |
| ESRD | DPP4i vs SU | 0.06 | -0.02 | 0.14 |
|  | SGLT2i vs SU | -0.81 | -0.89 | -0.73 |
|  | SGLT2i vs DPP4i | -0.87 | -0.95 | -0.79 |

#### **Figure A9:** Figure showing the mean difference in the predicted mean values for HbA1c (%), eGFR (mL/min/1.73m2), SBP (mm Hg), and BMI (kg/m2), compared across counterfactual second-line oral antidiabetic treatment scenarios by CVD status. The solid lines show the mean difference and the dotted lines the 95% confidence interval. A change in HbA1c of ±0.2% is equivalent to ±2.2mmol/mol.


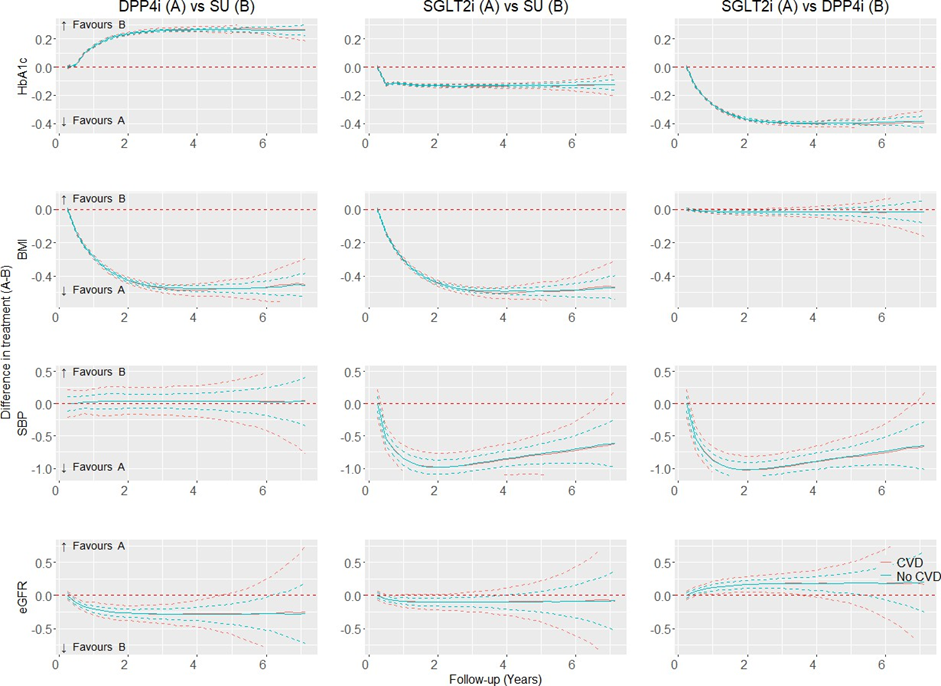


#### **Figure A10:** Figure showing the mean difference in the predicted mean values for HDL (mg/dL), LDL (mg/dL), total cholesterol (mg/dL), triglycerides (mg/dL), and DBP (mm Hg), compared across counterfactual second-line oral antidiabetic treatment scenarios by CVD status. The solid lines show the mean difference and the dotted lines the 95% confidence interval.


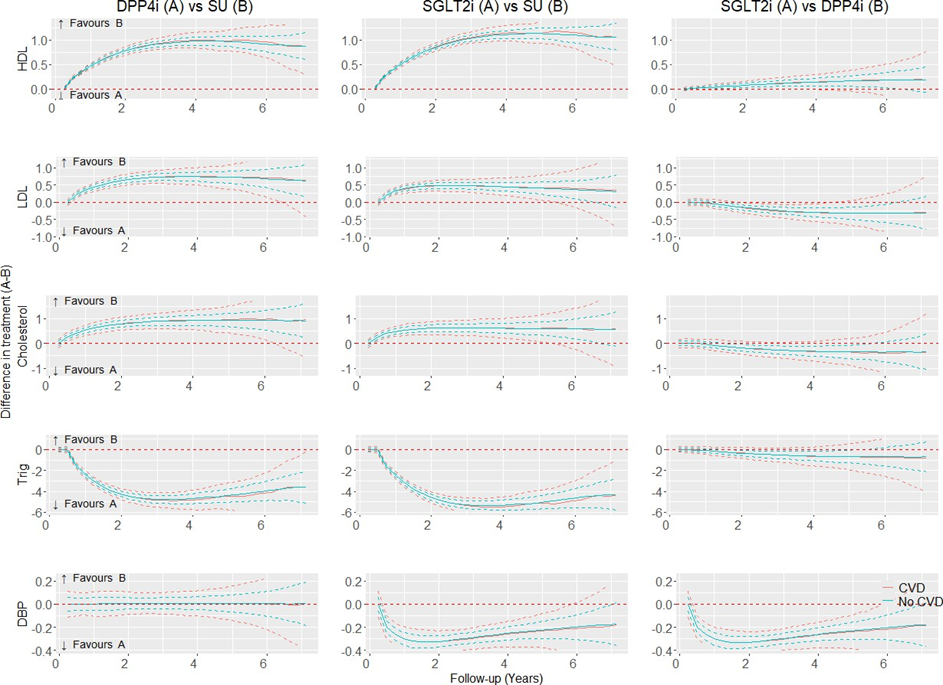


#### **Figure A11:** Figure showing the mean difference in the predicted probability for ESKD, MI, LE amputation, and HF, compared across counterfactual second-line oral antidiabetic treatment scenarios by CVD status. The solid lines show the mean difference and the dotted lines the 95% confidence interval.


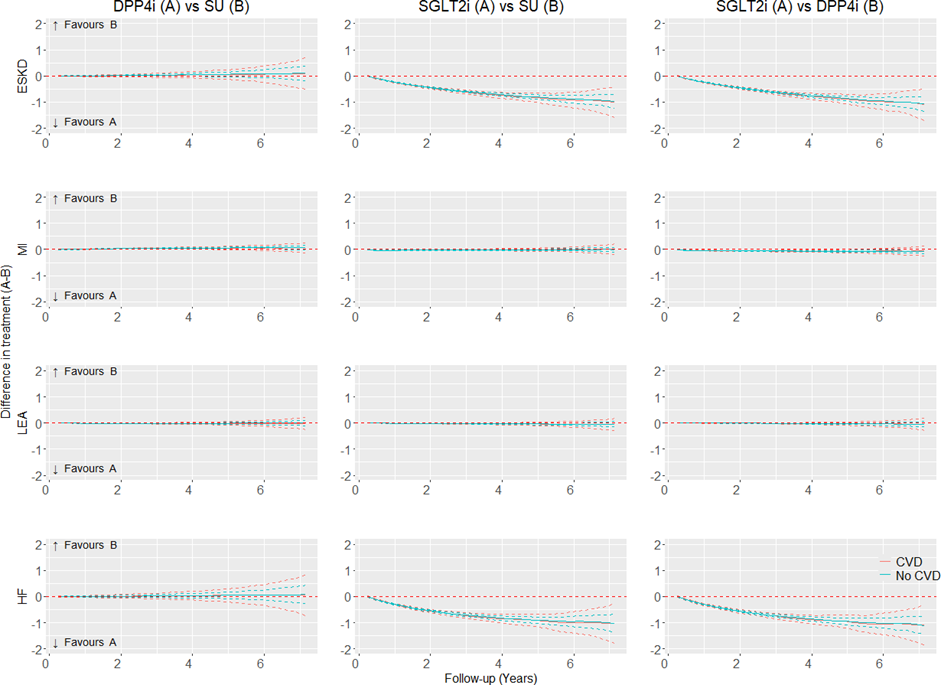


#### **Figure A12:** Figure showing the mean difference in the predicted probability for all-cause death, angina, stroke, diabetic eye disease, and hypoglycaemia, compared across counterfactual second-line oral antidiabetic treatment scenarios by CVD status. The solid lines show the mean difference and the dotted lines the 95% confidence interval.


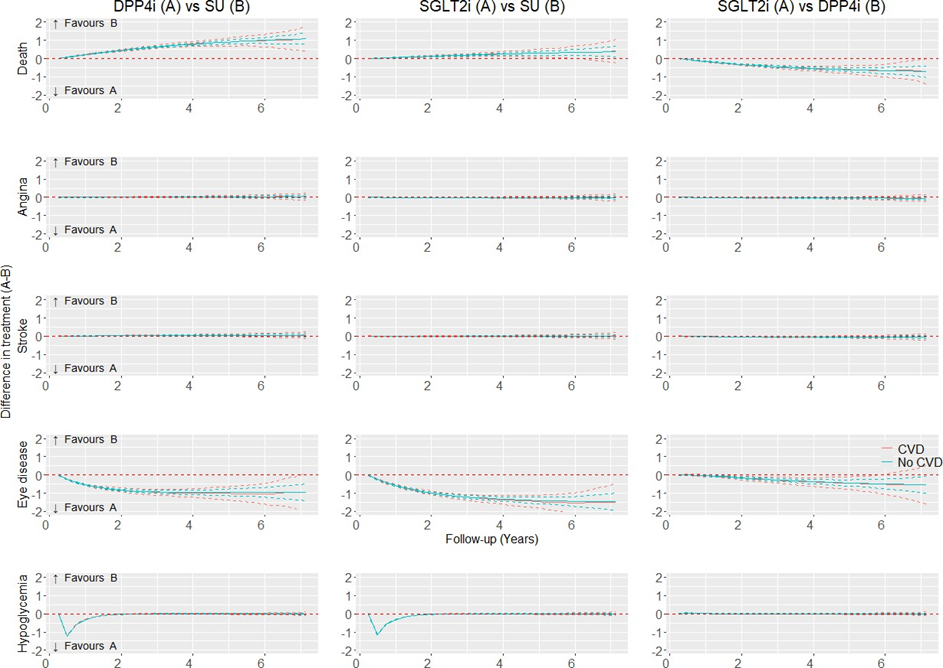


# **References**

1. Bidulka P, Lugo-Palacios DG, Carroll O, et al. Comparative effectiveness of second line oral antidiabetic treatments among people with type 2 diabetes mellitus: emulation of a target trial using routinely collected health data. *BMJ (Clinical research ed)*. 2024;385:e077097. doi:10.1136/bmj-2023-077097

2. Willan AR, Lin DY, Cook RJ, Chen EB. Using inverse-weighting in cost-effectiveness analysis with censored data. *Stat Methods Med Res*. Dec 2002;11(6):539-51. doi:10.1191/0962280202sm308ra

3. Basu A, Montano-Campos F, Huang ES, Laiteerapong N, Barthold D. Updating and calibrating the Real-World Progression In Diabetes (RAPIDS) model in a non-Veterans Affairs population. *Diabetes, obesity & metabolism*. Nov 2024;26(11):5261-5271. doi:10.1111/dom.15878

4. Terza JV, Basu A, Rathouz PJ. Two-stage residual inclusion estimation: Addressing endogeneity in health econometric modeling. *Journal of Health Economics*. 2008/05/01/ 2008;27(3):531-543. doi:<https://doi.org/10.1016/j.jhealeco.2007.09.009>

5. Basu A, Sohn M-W, Bartle B, Chan KCG, Cooper JM, Huang E. Development and Validation of the Real-World Progression in Diabetes (RAPIDS) Model. *Medical decision making : an international journal of the Society for Medical Decision Making*. 2019;39(2):137-151. doi:10.1177/0272989X18817521

6. Maruthur NM, Tseng E, Hutfless S, et al. Diabetes Medications as Monotherapy or Metformin-Based Combination Therapy for Type 2 Diabetes: A Systematic Review and Meta-analysis. *Ann Intern Med*. Jun 7 2016;164(11):740-51. doi:10.7326/m15-2650

7. Alexander JT, Staab EM, Wan W, et al. The Longer-Term Benefits and Harms of Glucagon-Like Peptide-1 Receptor Agonists: a Systematic Review and Meta-Analysis. *Journal of general internal medicine*. Feb 2022;37(2):415-438. doi:10.1007/s11606-021-07105-9

8. Mazidi M, Rezaie P, Gao HK, Kengne AP. Effect of Sodium-Glucose Cotransport-2 Inhibitors on Blood Pressure in People With Type 2 Diabetes Mellitus: A Systematic Review and Meta-Analysis of 43 Randomized Control Trials With 22 528 Patients. *J Am Heart Assoc*. May 25 2017;6(6)doi:10.1161/jaha.116.004007

9. Musso G, Gambino R, Cassader M, Pagano G. A novel approach to control hyperglycemia in type 2 diabetes: sodium glucose co-transport (SGLT) inhibitors: systematic review and meta-analysis of randomized trials. *Ann Med*. Jun 2012;44(4):375-93. doi:10.3109/07853890.2011.560181

10. Karagiannis T, Paschos P, Paletas K, Matthews DR, Tsapas A. Dipeptidyl peptidase-4 inhibitors for treatment of type 2 diabetes mellitus in the clinical setting: systematic review and meta-analysis. *BMJ (Clinical research ed)*. Mar 12 2012;344:e1369. doi:10.1136/bmj.e1369

11. Glycemia Reduction in Type 2 Diabetes — Glycemic Outcomes. *New England Journal of Medicine*. 2022/09/22 2022;387(12):1063-1074. doi:10.1056/NEJMoa2200433

12. Oshima M, Jardine MJ, Agarwal R, et al. Insights from CREDENCE trial indicate an acute drop in estimated glomerular filtration rate during treatment with canagliflozin with implications for clinical practice. *Kidney international*. 2021/04// 2021;99(4):999-1009. doi:10.1016/j.kint.2020.10.042

13. Baigent C, Emberson J, Haynes R, et al. Impact of diabetes on the effects of sodium glucose co-transporter-2 inhibitors on kidney outcomes: collaborative meta-analysis of large placebo-controlled trials. *The Lancet*. doi:10.1016/S0140-6736(22)02074-8

14. McGuire DK, Shih WJ, Cosentino F, et al. Association of SGLT2 Inhibitors With Cardiovascular and Kidney Outcomes in Patients With Type 2 Diabetes: A Meta-analysis. *JAMA Cardiol*. Feb 1 2021;6(2):148-158. doi:10.1001/jamacardio.2020.4511

15. Brookhart MA, Schneeweiss S. Preference-based instrumental variable methods for the estimation of treatment effects: assessing validity and interpreting results. *The international journal of biostatistics*. 2007;3(1):Article 14. doi:10.2202/1557-4679.1072

16. Basu A. ESTIMATING PERSON-CENTERED TREATMENT (PeT) EFFECTS USING INSTRUMENTAL VARIABLES: AN APPLICATION TO EVALUATING PROSTATE CANCER TREATMENTS. *J Appl Econ (Chichester Engl)*. June/July 2014;29(4):671-691. doi:10.1002/jae.2343

17. Martínez-Camblor P, Mackenzie T, Staiger DO, Goodney PP, O'Malley AJ. Adjusting for bias introduced by instrumental variable estimation in the Cox proportional hazards model. *Biostatistics*. Jan 1 2019;20(1):80-96. doi:10.1093/biostatistics/kxx062
